# Early Family Life Course Standardization in Sweden: The Role of Compositional Change

**DOI:** 10.1007/s10680-019-09551-y

**Published:** 2020-01-13

**Authors:** Zachary Van Winkle

**Affiliations:** Department of Sociology and Nuffield College, University of Oxford, 42-43 Park End Street, Oxford, OX1 1DJ UK

**Keywords:** Family, Life course, Sequence analysis, Educational expansion, Social change

## Abstract

The diversity of early family life courses is thought to have increased, although empirical evidence is mixed. Less standardized family formation is attributed to compositional changes in educational attainment, labour market participation, and childhood living conditions. I investigate whether and why family trajectories have become more or less standardized across birth cohorts in Sweden. I combine sequence metrics with Oaxaca–Blinder decompositions to assess the compositional shifts that drive changes in family formation standardization. Family trajectories of individuals born in 1952, 1962, and 1972 from age 18 to 35 are reconstructed using Swedish register data. My results demonstrate that early family formation has become more standardized across birth cohorts. Further, compositional differences between birth cohorts partially account for this standardization, especially for women. For example, higher levels of educational attainment are associated with family formation standardization. This substantiates arguments that family formation may re-standardize following the second demographic transition.

**Electronic supplementary material:**

The online version of this article (10.1007/s10680-019-09551-y) contains supplementary material, which is available to authorized users.

## Introduction

Many scholars have claimed that early family life courses have become more diverse across European societies starting in the mid-twentieth century (see Buchmann and Kriesi 2011). Young adults leave the parental home, marry, and enter parenthood at later ages, and some never enter marriage or parenthood (Goldscheider 1997; Rowland 2007). Additionally, cohabitation (Heuveline and Timberlake 2004), single parenthood (Heuveline et al. 2003), divorce (Schoen and Canudas-Romo 2006), and remarriage (Coleman et al. 2000) have become more common. In sum, life course patterns tend to characterize increasingly smaller portions of populations, which results in less standardized family life courses (Brückner and Mayer 2005).

It is important to study family life course de-standardization, because increasing diversity may have serious consequences for individuals and societies (see Zimmermann and Konietzka 2017 for a discussion). High life course diversity that is generated by nonmarital parenthood, serial cohabitation, and divorce is likely tightly intertwined with the production of social inequalities and their reproduction across generations (e.g. McLanahan and Percheski 2008). Further, more diverse family life courses are associated with increasing unpredictability, which may affect individuals’ ability to plan their lives, e.g. when to enter parenthood. Moreover, de-standardization poses challenges to social policies that aim to maintain the economic and subjective well-being of individuals with increasingly distinct family trajectories.

There are, however, two gaps in the empirical literature on the de-standardization of family life courses. First, findings are mixed as to whether a de-standardization has even occurred. For example, while Elzinga and Liefbroer (2007) report that family life courses had become more diverse across cohorts within a number of countries using the Family and Fertility Survey, Zimmerman and Konietzka (2017) observe a decrease in diversity following an initial increase in several Generations and Gender Programme countries. This is in line with Huinink’s (2013) proposition that a re-standardization of family lives may occur if de-standardization is the result of one dominant family pattern replacing another. Second, most studies on changing family life courses estimate associations between individual level characteristics, such as education, and individual trajectories (e.g. Van Winkle 2018). However, no research has quantified the extent that micro-level associations translate into population-level change. Both associations between individual characteristics and family trajectories as well as compositional change in those individual characteristics are needed to induce a shift in family life course standardization.

This study takes a first step to address these gaps with two research questions: (1) have early family life courses become more or less standardized across birth cohorts in Sweden, and (2) to what extent can the increase or decrease in early family life course standardization be attributed to compositional differences between birth cohorts. Sweden is an ideal case for these questions for two reasons: First, I should be to observe whether family formation has re-standardized following a shift in life course patterns, because dramatic changes in family demographic behaviour that were first observed are now thought to have stabilized (Ohlsson-Wijk 2011). Second, Swedish society has undergone extensive compositional change during the same period. Women’s educational attainment and labour market participation has increased dramatically across birth cohorts. Further, material living conditions during childhood also increased rapidly across the twentieth century.

One of my contributions to the family sociological and demographic literature is to propose methodological approach that combines metrics developed in sequence analysis with econometric decomposition methods to estimate and account for change in family life course standardization. The family trajectories of men and women born in 1952, 1962 and 1972 from age 18 to 35 are reconstructed with information from Swedish birth, civil status, residence, and death registries. I concentrate on ages 18–35, because this is thought to be the most demographically dense phase in the life course (Cook and Furstenberg 2002). I measure family life course standardization as the average of normalized sequence distances, which indicates how dissimilar an individual’s family trajectory is compared to everyone else within the same birth cohort. This measure allows me to compare birth cohort averages as an indicator of whether family life courses have become more or less standardized. Further, this metric can be used in Oaxaca–Blinder decompositions (Blinder 1973; Oaxaca 1973) to estimate whether and to what extent compositional change across birth cohorts is associated with change in family life courses. Specifically, I examine the extent that shifts in educational attainment, labour market participation, parental resources, and childhood family structure are associated with changing levels of family life course standardization.

This study also makes an empirical contribution that has theoretical implications for the comparative literature on family formation and the transition to adulthood. First, I demonstrate that contrary to common conceptions, early family life courses have become more standardized across birth cohorts in Sweden. This is the result of a transitory shift, where patterns of early marriage and parenthood have been replaced by patterns of late parenthood within cohabitation often followed by marriage. Second, I show that compositional changes in childhood family structure, parental resources, and educational attainment between birth cohorts partially account for the standardization of family life courses in Sweden.

## Theoretical Background

In the following sections, I first discuss compositional shifts in Sweden, such as changes in childhood living standards, educational attainment, and family demographic behaviour. Next, I conceptualize family life course change and, based on the discussion of compositional shifts in family formation, argue that life courses have become more standardized as delayed entry into parenthood within cohabitation has become a new dominant life course pattern. In a third section, I briefly sketch theories that link individuals’ social background, educational attainment, and childhood family structure to their family demographic behaviour at the micro-level. For example, numerous theoretical approaches associate high socio-economic background, educational attainment, and labour market attachment with delayed marriage and parenthood. Finally, I link compositional shifts to change in family life courses. Specifically, I argue that early family life courses have become more standardized as childhood standards of living as well as levels of educational attainment and labour market attachment have increased. However, I also highlight arguments that changes in childhood family structure may have polarized early family life courses, leading to lower levels of life course standardization.

### The Swedish Context from 1960–2010

Swedish labour market institutions and family policies have traditionally aimed at securing individuals from unemployment as well as maintaining high female labour market participation and high fertility rates. However, Swedish society and its institutions have undergone great changes since the 1960s. An overview of education, family, and labour market statistics associated with these changes is displayed in Table 1. As in most European countries, the second half of the twentieth century is associated with a considerable expansion of the tertiary education system. Only four tertiary education institutions existed in Sweden in 1960. However, 17 new universities were founded following a reform in 1974 (Andersson et al. 2004). Although university enrolment rates grew relatively slowly at first, between 1980 and 2010 the percentage of the population with a tertiary education grew from 14.4 to 25.2%.

**Table 1:** An overview of education, family, and labour market changes in Sweden between 1960 and 2010

	1960	1970	1980	1990	2000	2010
Population with university education^3^	5.9	7.4	14.4	18.8	23.1	25.2
Total fertility rate^1^	2.17	1.94	1.68	2.14	1.55	1.98
Nonmarital births (as per cent of live births)	11.3	18.6	39.7	47.0	55.3	54.2
Average age at marriage^1^						
Men	27.3	26.2	29.0	30.2	33.2	35.5
Women	24.3	24.0	26.4	27.6	30.6	32.9
Crude marriage rate^2^	6.7	5.4	4.5	4.7	4.5	5.3
Crude divorce rate^2^	1.2	1.6	2.4	2.3	2.4	2.5
Female labour force participation^1,3^	63.4	59.4	74.2	80.9	75.0	77.8
Unemployment rate^3^	1.7	1.5	2.2	1.8	5.8	8.3
Average income^1^ (in 1000 2017 SEK)	109.4	138.7	151.9	157.8	208.1	265.5
Net household income inequality (Gini)^3^	0.27	0.25	0.20	0.17	0.25	0.23

^1^Information provided from the annual statistical abstracts published by Statistics Sweden (see https://www.scb.se/en_/Finding-statistics/Historical-statistics/Statistical-Yearbook-of-Sweden/ and https://www.scb.se/hitta-statistik/statistik-efter-amne/ovrigt/ovrigt/statistisk-arsbok/)

^2^Information provided by EuroStat (see https://ec.europa.eu/eurostat/statistics-explained/index.php/Marriage_and_divorce_statistics)

^3^Infromation provided from the Comparative Welfare State Database (see David Brady, Evelyne Huber, and John D. Stephens, Comparative Welfare States Data Set, University of North Carolina and WZB Berlin Social Science Center, 2014)

The Swedish labour market and labour market institutions were surprisingly stable between 1960 and 1990. Labour market participation rates remained high and increased, and unemployment remained under 3%. Further, real wages rose consistently and income inequality decreased. These developments have been largely attributed to highly centralised wage bargaining, high union density, and extensive activating labour market policies (Edin and Topel 1997; Van Winkle and Fasang 2017). However, the Swedish labour market was hit by a major recession in the 1990s, which increased pressure for labour market reforms. Unemployment and income inequality increased as a result of the recession. Nonetheless, absolute incomes increased from 157,800 Swedish Krona (SEK) in 1990 to 265,500 SEK in 2010, partially due to a more educated workforce.^1^

Swedish family policy is perceived to be among the most family friendly. Since 1963 Swedish mothers benefited from more than 26 weeks of paid and job protected maternity leave. In 1974 maternity leave was replaced by an extensive parental leave system, which by 1980 was expanded to one year of paid and job protected leave to be split between mothers and fathers (Ifo-Insitute 2015). Further, a 10-day paternity leave was introduced in 1980. Since the mid-1990s, publicly subsidised early and preschool childcare became widely available (Garrouste 2010). Nonetheless, fertility and marriage became less common across the late twentieth century, accompanied by increases in divorce and nonmarital childbirth. The total fertility rate dropped from 2.17 in 1960 to 1.55 in 2000. Further, the crude marriage rate decreased from 6.7 in 1960 to 4.5 in 2000. Following the introduction of no-fault divorce legislation in 1974, the crude divorce rate doubled from 1.2 in 1960 to 2.4 in 1980. However, both marriage and divorce rates stabilized following the 1980s to roughly 4.5 and 2.4, respectively. Despite relatively stable marriage rates, nonmarital childbirths continued to increase. By the year 2000, over half of all live births occurred outside of marriage, although the vast majority to cohabiting couples.

### Conceptualizing Early Family Life Course Standardization

Age 16–35 is thought to be the most demographically dense phase in the life course. Cook and Furstenburg (2002) demonstrated that despite cross-national differences in the timing and order of life course events, most individuals have completed full-time education, entered full-time employment, founded an independent household, and have married and entered parenthood by age 35. The timing of marriage and parenthood has traditionally been the focus of demographic and sociological research on early family life courses (Hogan and Astone 1986). However, more recent research has focused on other early life course events that are associated with marriage and parenthood, such as parental home leaving, cohabitation, and divorce (Buchmann and Kriesi 2011). This is important, because it has enabled scholars to study more comprehensive life course patterns across socio-historic contexts.

The extent that family life courses vary within populations is affected by three factors: variation in (1) the occurrence of family life course states, (2) the timing of transitions between two states, and (3) the order of states in the life course (Huinink 2013). For example, early family life courses in the early twentieth century are thought to have varied only slightly (Mayer 2004). Early family life courses consisted of two tightly coupled events, marriage and parenthood. However, early family life courses in the late twentieth century are thought to vary to a much greater extent. The number of family states has increased, and the timing and order of transitions has changed. For example, individuals tend to enter cohabitation at relatively young ages, while the transition to parenthood often occurs much later. Further, marriage no longer precedes parenthood, but is often its antecedent. Therefore, it is important to study family life courses holistically as “process outcomes” (Abbott 2005), because life courses vary in the occurrence, timing, and order of states and transitions as well as the duration spent within states. Process outcomes, such as family trajectories, can be conceived as the result of numerous “point-in-time outcomes”, such as the timing of first and subsequent births.

Recent literature contends that family life courses variation has increased, although the empirical evidence is mixed (Van Winkle 2018). Terms denoting this increase in variation are used ambiguously (Brückner and Mayer 2005). De-standardization is most common and signifies an increase in life course patterns that diverge from early marriage and parenthood (Brüderl 2004) or an increase in family formation dissimilarity relative to early marriage and parenthood (Hofäcker and Chaloupková 2014). However, an increase in family patterns that differ from trajectories of early marriage and parenthood is better described as de-traditionalization or de-institutionalization (Kohli 1985). Rather than a shift away from traditional life course patterns, Brückner and Mayer (2005) define de-standardization as a process in which patterns characterize smaller portions of a population or in which life course events occur at more dispersed ages. Therefore, standardization is a process where life course patterns characterize larger proportions of the population.

It is most commonly assumed that *early family life courses have become less standardized across birth cohorts (H1)* (see column 1 of Table 2). This is generally founded on evidence that early marriage followed by parenthood has become less common and has been replaced by diverse life course patterns (Corijn and Klijzing 2001). Next to lower rates of marriage and parenthood as well as postponed entry into marriage and parenthood, higher rates of cohabitation, divorce, and nonmarital childbirth are expected to decrease standardization levels.

**Table 2:** Overview of hypotheses

	H1	H2
Standardization levels	Increase	Decrease
*Compositional changes*		
A. Parental resources		X
B. Educational attainment		X
C. Labour market attachment		X
D. Childhood family structure	X	
H3 Gender difference	Stronger for women	Stronger for women

Family life courses may become more standardized if change is a transitory process where one dominant life course patterns replaces another (Huinink 2013). Indeed, the compositional shifts discussed in the previous section suggest that a shift in family life courses has occurred. Rather than early marriage followed by parenthood, the majority of individuals now enter parenthood within cohabiting unions at later ages (Baizán et al. 2004; Holland 2013). Moreover, divorce and marriage rates in Sweden stabilized in the 1980s rather than a continuation of declining trends in marriage and increasing trends in divorce (Ohlsson-Wijk 2011). Therefore, I find it more plausible that *early family life courses have become more standardized across birth cohorts (H2)* following a relatively universal shift in the timing and ordering of entering nonmarital cohabitation, parenthood, and marriage.

### Theories on Family Life Course Change

To develop hypotheses about which compositional shifts induced a standardization or de-standardization in early family life courses, it is necessary to review theories that link individual characteristics to family formation. Sociologists and demographers have developed numerous theoretical approaches to account for changes in the prevalence, timing, and ordering of family events. Generally, these can be subsumed under four broad theoretical frameworks: an ideational, a rational choice, a structural constraint, and a socialization framework. The theoretical approaches within these frameworks commonly link individuals’ childhood standard of living, educational attainment, labour market attachment, and childhood family structure to their future family demographic behaviour.

The second demographic transition (SDT) thesis, one of the most prominent explanations within the ideational framework, has a long history of relating cultural change to a de-standardization of family life courses (see Zaidi and Morgan 2017 for a review). The core argument of the SDT thesis postulates that as childhood standards of living increase, individual value orientations will shift to prioritize self-actualization rather than the long-term commitments of early marriage and parenthood (Lesthaeghe 2010, 2014). This in turn initiates an irreversible increase in the mean age at marriage and parenthood, a lower propensity to marry and enter parenthood, as well as a higher prevalence of singlehood and cohabitation, divorce, and nonmarital childbirth.

Other theoretical explanations within the ideational framework concentrate on the relationship between educational attainment, gender norms and family formation (Goldscheider 2000; e.g. McDonald 2000). Recently, Goldscheider et al. (2015) predicted that fertility rates and marital stability will increase when gender-egalitarian norms become more widespread and men’s gender roles adapt to women’s. Esping-Andersen and Billari (2015) maintain that educational attainment is central to the spread of gender egalitarianism and the recuperation of fertility.

Prominent theoretical explanations within the rational choice framework also link childhood living standards and educational attainment to family formation. Family demographic decisions are described as utility maximization processes, where the utility of an event is a function of (opportunity) costs and benefits (e.g. Becker 1974a; Becker et al. 1977; Becker and Tomes 1994). Opportunity costs partially result from foregone income, rise with increases in an individual’s educational attainment and labour market experience. Higher educational attainment and labour market attachment should then lead to delayed or forgone marriage and parenthood. However, the opportunity costs associated with marriage and especially parenthood are highly context-contingent. In the Swedish context, with gender-egalitarian parental leave schemes and widespread public childcare, the opportunity costs of childbearing are likely much lower than in contexts with lower levels of public family support.

Other rational choice theorists have used a market framework to link living standards and educational attainment to the timing of marriage and parenthood. For instance, Easterlin (1975, 1976) predicted that couples will enter parenthood only after they had achieved their aspired standard of living developed during childhood. Oppenheimer (1988) used partner markets to account for variation in the timing of marriage. Specifically, she maintains that individuals spend time on the marriage market to find an acceptable match, which is partially a function of socioeconomic background. Men and women use the information currently available, e.g. educational attainment and labour market experience, to estimate the potential socioeconomic attainment of a possible partner. Further, she suggests that premarital cohabitation represents a cost-effective strategy to prolong the search for a future partner.

The structural constraint framework links globalization and deindustrialization to change in family life courses (Mills and Blossfeld 2003, 2005, 2013). Importantly, this framework suggests that the mechanisms described by Easterlin (1975, 1976) and Oppenheimer (1988) will strengthen in times of increasing economic uncertainty. Young adults facing more precarious labour markets will tend to delay or forgo marriage and parenthood, and resort to other family life course models associated with fewer and shorter-term commitments, such as cohabitation. However, economic uncertainty will not affect all members equally, but will vary by socioeconomic background. Parents with high incomes and educations will invest in their children’s educational attainment and support their labour market entry to diminish the risk of downward social mobility (Breen and Goldthorpe 1997; see also Bernardi and Grätz 2015; Erola and Kilpi-Jakonen 2017), which may lead to delayed marriage and parenthood. In sum, the economic uncertainty approach relates socioeconomic background to the timing and propensity of marriage and parenthood, but also to the occurrence of premarital cohabitation.

Finally, McLanahan’s (2004) diverging destinies hypothesis has brought more attention to the relationship between socialization and family formation. She contends that the socioeconomic and family outcomes of children from single-parent households are becoming increasingly less favourable compared to the outcomes of children from two-parent households (see also Martin 2000; Raver et al. 2015; Schoon 2015). Further, she maintains that this difference cannot be explained by socioeconomic differences between single- and two-parent households alone. The transmission of parents’ values and preferences, their ability to supervise and control their children’s activities, and stress generated by family instability are seen as important mechanisms associated with the intergenerational transmission family demographic behaviour (see Teachman 2003). While children from single-parent families are more likely to enter parenthood early and become single parents themselves, the children of two-parent families will delay parenthood until marriage.

### Compositional Shifts and Family Life Course Change

In the previous section, I described prominent theoretical approaches that link individual characteristics to the prevalence, timing, and ordering of family events. The theoretical frameworks and explanations discussed above are commonly depicted as separate approaches in a clear-cut manner; however, they may interact to generate population-level change in family life courses. In the following discussion, I will concentrate on how compositional shifts in childhood standards of living, educational attainment, labour market attachment, and childhood family structure across cohorts may stimulate change in family life courses through different theoretical pathways.

Higher childhood living standards and parental resources are of central importance to the ideational, rational choice, and structural constraints frameworks. In particular, the SDT thesis, Easterlin’s (1975, 1976) theory on fertility, Oppenheimer’s (1988) theory on marriage, and the structural constraints approach predict that higher parental education and incomes will induce more delayed entry into marriage and parenthood as well as lower rates of marriage and parenthood in times of economic uncertainty. Further, individuals from advantaged backgrounds will be more likely to postpone marriage and parenthood to pursue more individualistic life styles. An increased prevalence of premarital cohabitation may result from both a cost-effective strategy to prolong the search for an optimal match on the marriage market and as a new possible step in an individualistic life course.

A higher educated population has implications for gender-ideology theories within the ideational framework and utility theories within the rational choice framework. The opportunity costs related to marriage and parenthood increase as individuals, especially women, become more educated (Becker et al. 1977). Increased educational attainment is also associated with higher labour market attachment, which may additionally increase the opportunity costs of marriage and parenthood (e.g. Becker and Tomes 1994). A more educated population is more likely to hold gender-egalitarian norms, which increase gender equity within the private sphere (Esping-Andersen and Billari 2015; Goldscheider et al. 2015). As a result, marriage and fertility rates should stabilize, although individuals will likely continue to enter marriage and parenthood at later ages.

In sum, I expect that *higher levels of parental income and education as well as educational attainment and labour market experience are associated with higher levels of early family life course standardization (H2A, H2B, and H2C).* Increased standards of living, educational attainment, and labour market participation will delay entry into marriage and parenthood as well as increase cohabitation rates as individuals pursue more individualistic life styles, spend more time to establish themselves on the labour market and find an adequate partner, and fulfill their consumption aspirations to enter parenthood.

McLanahan’s (2004) diverging destinies framework stands out as the only approach that links a dramatic compositional shift, i.e. the number of single-parent households, with a de-standardization of family life courses. Specifically, a polarization in the transmission of family demographic behaviour between single-parent and two-parent families may lead to lower levels of life course standardization. While young adults from two-parent households enter parenthood within cohabitation at later ages, young adults from single-parent households continue to enter marriage and parenthood at early ages. This process may also be reinforced by the associations between childhood living standards and educational attainment discussed above: Single-parent households are on average less educated and have lower household incomes than two-parent families (Mood and Jonsson 2016). Further, children of single-parent families attain a lower level of education than children from two-parent families (Björklund et al. 2007). Both factors may additionally facilitate an earlier entry into marriage and parenthood (cf. Easterlin 1975, 1976; Oppenheimer 1988). In sum, *higher levels of single-parent families during childhood are associated with lower levels of early family life course standardization (H1D)*.

All the theoretical perspectives discussed above have one thing in common: they expect stronger associations for women compared to men. The frameworks proposed by Becker (1973, 1981) as well as Esping-Andersen and Billari (2015) explicitly state that increases in women’s educational attainment is the impetus for change in family life courses. The increase in educational attainment and labour market participation across birth cohorts in Sweden has been most pronounced for women. With regard to McLanahan’s (2004) diverging destinies framework, women are at a higher risk of becoming single custodial parents through lone birth or divorce. Therefore, parents may be especially interested in investing in their daughter’s educational and labour market attainment as well as influencing her partner choice. I therefore expect that *the associations between changing levels of educational attainment, labour market participation, childhood family structure, and parental resources with changing levels of early family life course standardization are stronger for women (H3)*.

## Data and Methods

### Sample and Sequence Definition

I use Swedish birth, civil status, residence, and death registries to reconstruct early family life courses for men and women born in 1952, 1962, and 1972.^2^ There are a total of 322,292 persons registered in the 1952, 1962, and 1972 birth registries. I exclude individuals that emigrated (19,478) or died (4770) before age 35, because I am not able to observe their family demographic behaviour until age 35. I also omit adopted individuals (5347) from the analyses to create a clear reference to those living with a single biological parent compared to two biological parents. Finally, persons are excluded if neither biological parent is registered (245) or if the individual cannot be linked to the multigenerational identification file (178). After those exclusions, I have a population of 98,914 persons born in 1952, 95,680 born in 1962, and 97,680 born in 1972.

Family trajectories are constructed as sequences with monthly information from age 18 to 35. Therefore, the sequences of the cohorts born in 1952, 1962 and 1972 are observed between ages 18 and 35 and cover the periods 1970–1987, 1980–1997 and 1990–2007 in historical time, respectively. At any given time, an individual can either be single (S), married (M) or divorced (D) with or without children (e.g. S or SC). Further, individuals can be cohabiting or separated with children (e.g. CC and SpC). Note that single (S) indicates that an individual has neither entered marriage nor parenthood, while single with children (SC) indicates that an individual has entered parenthood, but not within a marital or cohabiting union.

Unfortunately, I cannot identify parental home leaving or childless cohabitation in the Swedish registries. This could bias my results if the age variation of parental home leaving has increased or there is high variation in the prevalence of childless cohabitation. However, studies have demonstrated that individuals in Sweden leave the parental home at an early and universal age, and that childless cohabitation has become a relatively universal experience (e.g. Billari and Liefbroer 2010). The state separated indicates a separation following a cohabiting union and therefore cannot be identified without the presence of children. The data used in this study end in 2008, which is why the youngest birth cohort can only be followed until age 35. This truncation of life courses may limit the comparability of my birth cohorts if the timing of family formation events has shifted past the age of 35. However, many life course events still occur before age 35 for my youngest birth cohort. For example, both the mean age of marriage and first birth is between 29 and 31 for Swedish cohorts born in the 1970s (Billari and Liefbroer 2010). Cohorts born before 1952 could not be included in the analysis, because their parental incomes at age 16 cannot be linked in the registries. I discuss and test the influence that broader cohort specifications, the truncation of sequences at age 35, and not observing parental home leaving and childless cohabitation has on my results in the Robustness Checks section.

### Measuring Early Family Life Course Standardization

This study aims to assess whether family life courses have become more or less standardized and to ascertain which compositional shifts are associated with these changes. However, it is difficult to empirically assess the link between individual characteristics and family formation standardization, because the latter is a characteristic of populations. It is therefore necessary to measure standardization at the individual level. As discussed above, standardization implies that family life course patterns characterize larger segments of the population. At the individual level, this process manifests itself as individual life courses becoming more similar to one another. I therefore conceptualize standardization as a process in which the proportion of individuals with similar life courses compared to the life courses in their population increases. Conceptualizing standardization as increasing similarity has the advantage that there is no predetermined reference point. This is necessary to identify increasing family life course standardization that results from a transitory shift (Huinink 2013).

I use the average of normalized pairwise sequence distances to measure the similarity of family life courses. This metric reflects life course dissimilarity within populations (Zimmermann 2013; Fasang 2014) and has been shown to be suitable for comparisons across birth cohorts (Studer and Elzinga 2016). It is calculated in three steps: (1) a pairwise sequence distance matrix is generated, (2) sequence distances are normalized to represent dissimilarities, and (3) dissimilarities are averaged across members of the same birth cohort. I calculate pairwise sequence distances using optimal matching (OM) (MacIndoe and Abbott 2004), which is defined as the minimum cost needed to transform one sequence into another. Formally, the OM distance, *d*_OM_, between sequence *x* and *y* is the minimum cost of the edits, *C*(*e*)*,* to transform sequence *x* into sequence *y*:

1.1 $${d}_{\text{OM}}\left(x,y\right)=\min\left\{C\left(e\right):e\in E\left(x,y\right)\right\},$$

where *E*(*x*,* y*) represent a series of substitution, deletion and insertion edits (Elzinga 2014). The results displayed below are calculated using insertion and deletion costs of 2 and constant substitution costs of 2. The results are generally robust to other distance metrics. The pairwise distances are then normalized using an empty reference sequence, *r*, that allows the comparison of scales and transforms distances into dissimilarities:

1.2 $${d}_{r}=\frac{{d}_{\text{OM}}\left(x,y\right)}{\left[{d}_{\text{OM}}\left(x,y\right)+{d}_{\text{OM}}\left(x,r\right)+{d}_{\text{OM}}\left(y,r\right)\right]/2}.$$

It is important to normalize distances to allow comparisons across birth cohorts, but especially to have a measure that reflects similarity (Elzinga 2014). The indicator for a respondent’s family life course dissimilarity, the average of normalized pairwise sequence distances, ${\bar{d}}_{{r}_{i}}$, is calculated as the sum of a respondent’s normalized distances divided by the cohort’s respective number of observations:

1.3 $${\bar{d}}_{{r}_{i}}=100\times \frac{1}{n}\sum_{j=1}^{J}{d}_{{r}_{i,j}},$$

where ${d}_{{r}_{i,j}}$ denotes the normalized distance of the respondent’s family sequence, *i*, to the family sequence of another respondent, *j*. Thus, small values indicate that a respondent’s sequence is similar to all the other sequences in their respective cohort, while large values indicate a dissimilar sequence. This dissimilarity measure can be interpreted as the average cost of edits multiplied by 100 needed to transform an individual’s sequence into another sequence within his or her birth cohort. For the sake of simplicity and readability, I refer to average normalized sequence distance as dissimilarity and the average cost of edits multiplied by 100 as the cost of edits in the following sections.^3^

As the time needed to calculate sequence distance increases exponentially with sample size, I draw a random sample of 10,000 individuals from the population of each study cohort and construct sequences. Individuals are excluded if their sequences cannot be constructed due to missing information, usually if it cannot be determined if an individual is cohabiting with the child’s mother or father after transitioning into parenthood. This leaves me with 9565 sequences for the 1952 cohort, 9513 sequences for the 1962 cohort and 9,840 sequences for the 1972 cohort, 95.6%, 95.1%, and 98.4% of the 1952, 1962, and 1972 cohort random draw, respectively.

### Independent Variables

I use information from the quinquennial national household censuses and the longitudinal integration database for health insurance and labour market studies (LISA) for individual’s educational attainment at age 35. Educational attainment is measured in three categories for comparability across cohorts: years of schooling associated with lower-secondary schooling, upper-secondary schooling, or more then upper-secondary schooling. Labour market participation is calculated as the number of years an individual reported labour market income between age 18 and 35 in the income and tax registry or in LISA.

The Swedish multigenerational register enables researchers to link parents and children in the registers. This allows me to use information from the registries to generate variables for childhood family structure and parental resources. Data on co-residence and marriage are used to identify whether children lived with both biological parents until they were age 16 or whether they lived with a single parent at any time between birth and age 16 (Thomson and Eriksson 2013). I drop individuals if the residence of either biological parent is not registered at any time between birth and age 16, which means that individuals are excluded if either biological parent dies during childhood. This is important to ensure that the childhood family structure variable captures single parenthood through lone birth or through separation, because the effects of parental death on life course outcomes are known to differ considerably (Biblarz and Gottainer 2000).

I use mother’s and father’s educational attainment and total income when individuals were age 16. Parental education is defined using the dominance principle, meaning that the highest education level of either parent is used. Parent’s educational attainment is also taken from the national household censuses and LISA and includes a fourth educational category: whether parents did not complete lower-secondary school. I measure parental income as the total earned income of both parents reported in the income and tax registers when individuals where age 16. Income is included in the analyses in steps of 100,000 SEK, which corresponds to approximately 12,300 US dollars. I exclude individuals if both parents are missing information on education or income. Mother’s age at first birth, which is generated by linking mother’s birth registry information to their children, is controlled to adjust for higher parental incomes that are attributable to age differences. After listwise deletion of observation with missing information on any independent variables, I retain 7980 (83.4%), 9386 (98.6%), and 9799 (99.5%) individuals from the 1952, 1962, and 1972 samples, respectively, used to calculate sequence distance. Summary statistics on the population, the sample used to calculate sequence distances, and the analysis sample, are displayed in the online supplement by birth cohort (see Table 1, section VII). A comparison shows that there are no substantial differences between the population and the samples that might induce selectivity bias.

### Decomposing the Dissimilarity Differential

I propose using Oaxaca–Blinder decompositions (Blinder 1973; Oaxaca 1973) to ascertain whether change in family life course standardization across cohorts can be attributed to compositional change. Oaxaca–Blinder decompositions are widely used in labour market economics and sociology to decompose income differences between groups, such as men and women, into “explainable” and “unexplainable” portions (Stanley and Jarrell 1998; Weichselbaumer and Winter-Ebmer 2005). The “explainable” portion of an income gap is the differential that results from group differences in individual characteristics, such as educational attainment and work experience. The “unexplainable” portion is the differential that results from group specific associations between individual characteristics and income, e.g. group specific education coefficients.

Oaxaca–Blinder decompositions decompose mean differences in a counter-factual manner into (1) a “composition component” representing the differential due to mean differences in regressor predictors and (2) an “association component” representing the differential due to group specific regression coefficients. Formally, the overall difference in the means between two groups *A* and *B*, *Δ*_*O*_, can be estimated as the difference between group specific linear predictions from a vector **X** consisting of predictor variables and their slope coefficients contained in **ß**:

2.1 $${\varDelta }_{O}=E\left({Y}_{A}\right)-E\left({Y}_{B}\right)=E{\left({X}_{A}\right)}^{^{\prime}}{\beta }_{A}-E{\left({X}_{B}\right)}^{^{\prime}}{\beta }_{B}.$$

Assuming the conditional mean of the residuals is zero, the overall difference can be written as the differences in predictor means weighted by group *B*’s coefficients and the differences in coefficients weighted by group *A*’s predictor means:

2.2 $${\varDelta }_{O}={\left[E\left({X}_{A}\right)-E\left({X}_{B}\right)\right]}^{^{\prime}}{\beta }_{B}+E{\left({X}_{A}\right)}^{^{\prime}}\left({\beta }_{A}-{\beta }_{B}\right).$$

Or simply:

2.3 $${\varDelta }_{O}={\varDelta }_{\text{Composition}}+{\varDelta }_{\text{Association}},$$

where the composition component, *Δ*_Composition_, describes the expected change for group *B*’s mean outcome given group *A*’s predictor means, and the association component, *Δ*_Association_, describes the expected change given group *A*’s coefficients. Commonly the more productive or non-discriminatory structure has been used in the economic and sociological literature when explaining income differences, such as men’s coefficients rather than women’s (Oaxaca 1973). Others have used a scalar matrix with weighted sample proportions (Cotton 1988), OLS estimates from pooled samples without controlling for group membership (Neumark 1988), and estimates from a pooled model adjusted for group membership (Jann 2008). The results below use the latter method, because it represents a structure independent of cohort membership and there is no justification for assuming one birth cohort should serve as a reference structure.

Combining the sequence dissimilarity measure and Oaxaca–Blinder decompositions allows me to establish a micro–macro-link between individual characteristics and population level standardization. The overall mean difference in dissimilarity between two birth cohorts, as formulated in Eq. 2.1, indicates whether family life course has become more or less standardized across birth cohorts. Hypothesis H1 that family life courses have become less standardized is supported if average dissimilarity increases. If average dissimilarity decreases, then hypothesis H2 is supported.

The decomposition of the mean difference in dissimilarity gives insight into the extent that compositional changes are associated with changes in family life course standardization. As expressed in Eqs. 2.2 and 2.3, I estimate what the overall difference in dissimilarity between two cohorts would have been without compositional change. Further, a detailed decomposition indicates how influential specific individual characteristics are in driving changing levels of standardization. For example, what the overall difference in dissimilarity would have been without differences in educational attainment. For simplicity, I display only the total detailed components for parental and individual educational attainment rather than the level-specific components, e.g. post-secondary education (Oaxaca and Ransom 1999). I do not discuss the association components of the Oaxaca–Blinder decomposition, because I am primarily interested in the association between compositional shifts and change in family formation standardization. However, results from OLS regressions on dissimilarity and the detailed association components are displayed by birth cohort in the manuscript appendix (see Tables 5, 6).

In the following section, I first describe the qualitative changes in family formation across birth cohorts through sequence visualization to gain a deeper understanding of the processes that lie behind the decomposition results. Second, I discuss compositional changes in average dissimilarity and individual characteristics across birth cohorts. Finally, I estimate Oaxaca–Blinder decompositions on the dissimilarity differentials between 1952–1962, 1952–1972, and 1962–1972. Sequence distances and therefore average dissimilarity is calculated on a combined sample of men and women. I also visualize men and women’s sequences together. However, I calculate means, discuss compositional differences, and estimate Oaxaca–Blinder decompositions separately by gender to test my hypothesis H3 that the composition components are stronger for women compared to men.

## Results

### Qualitative Changes in Early Family Life Courses

**Fig. 1:**
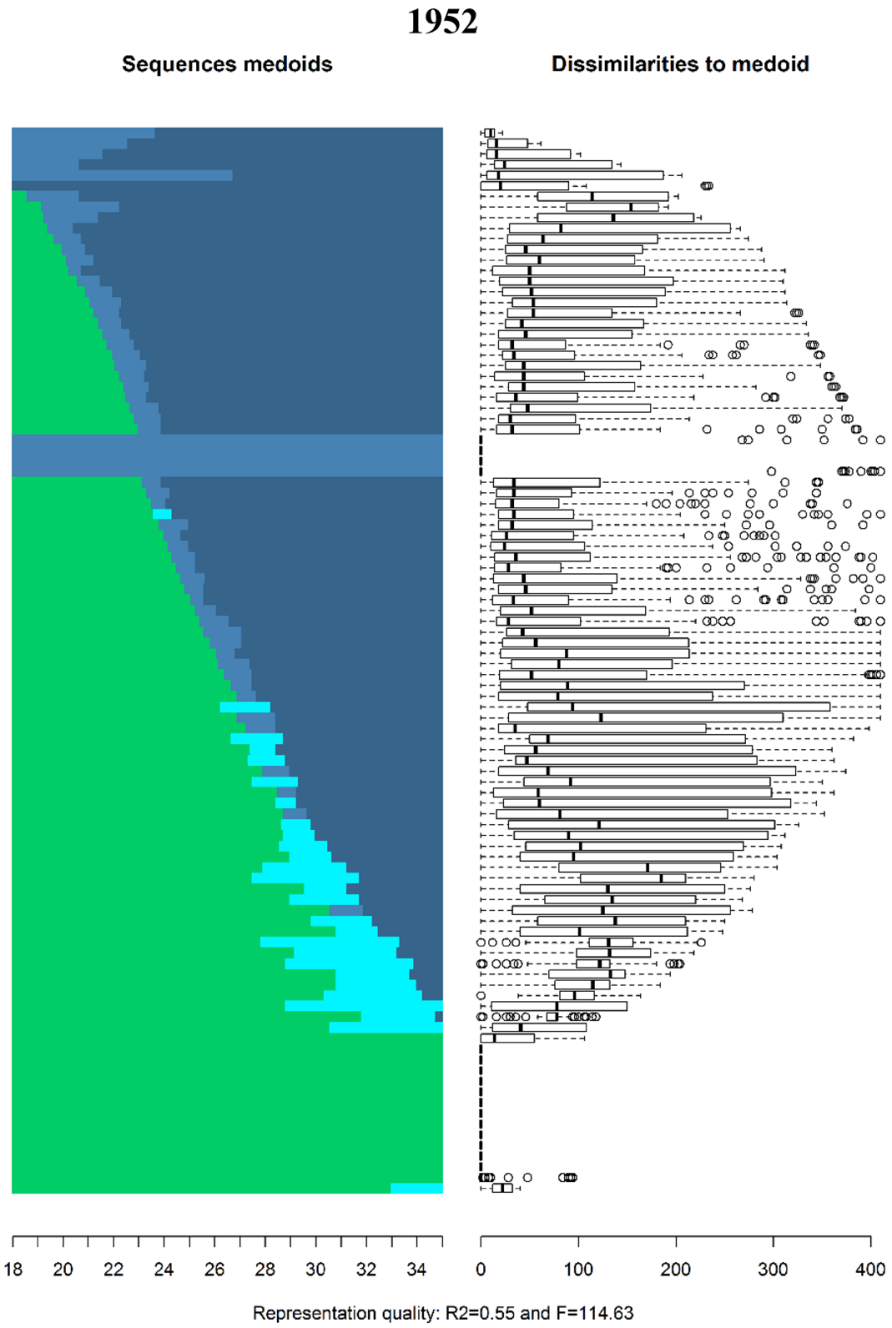

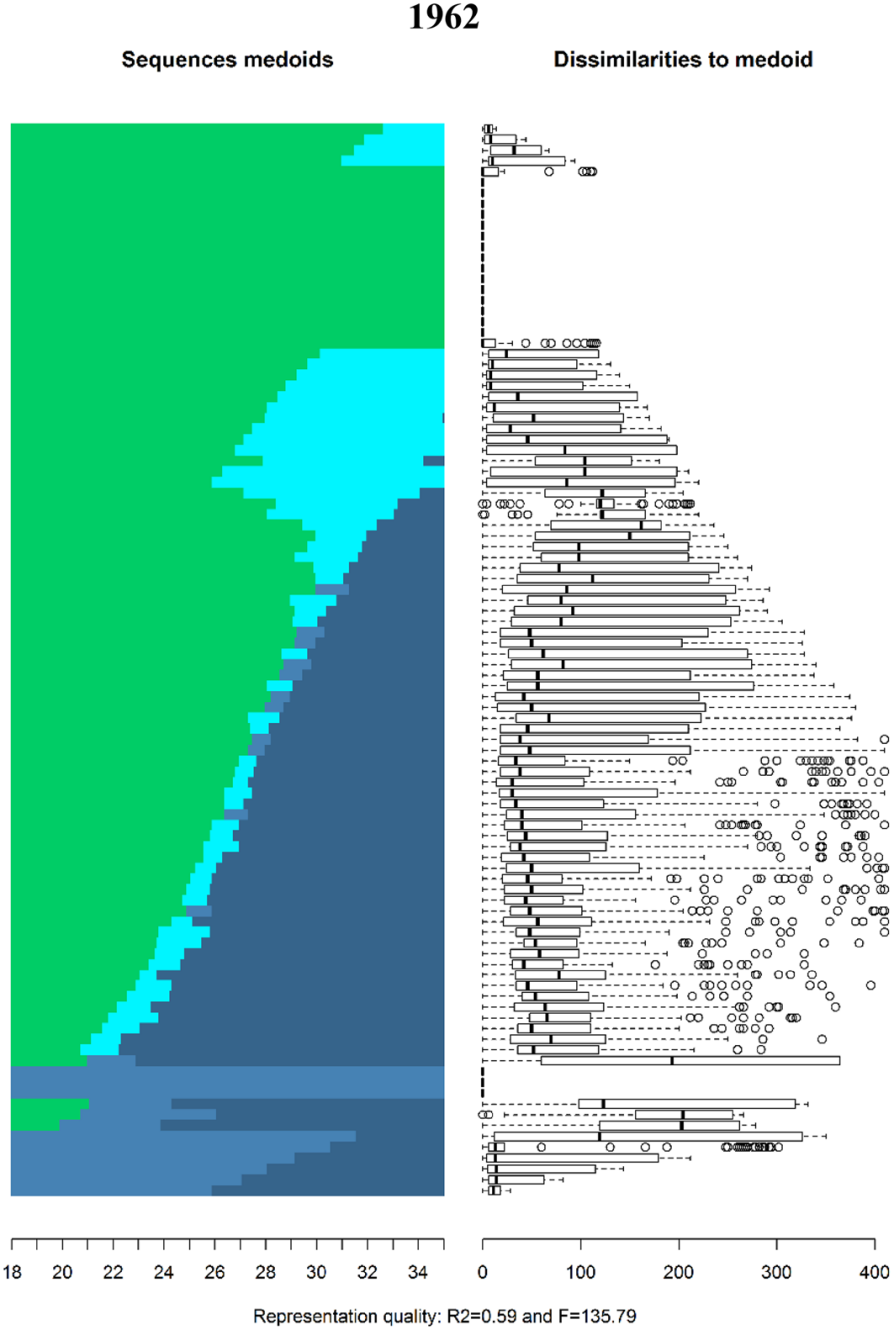

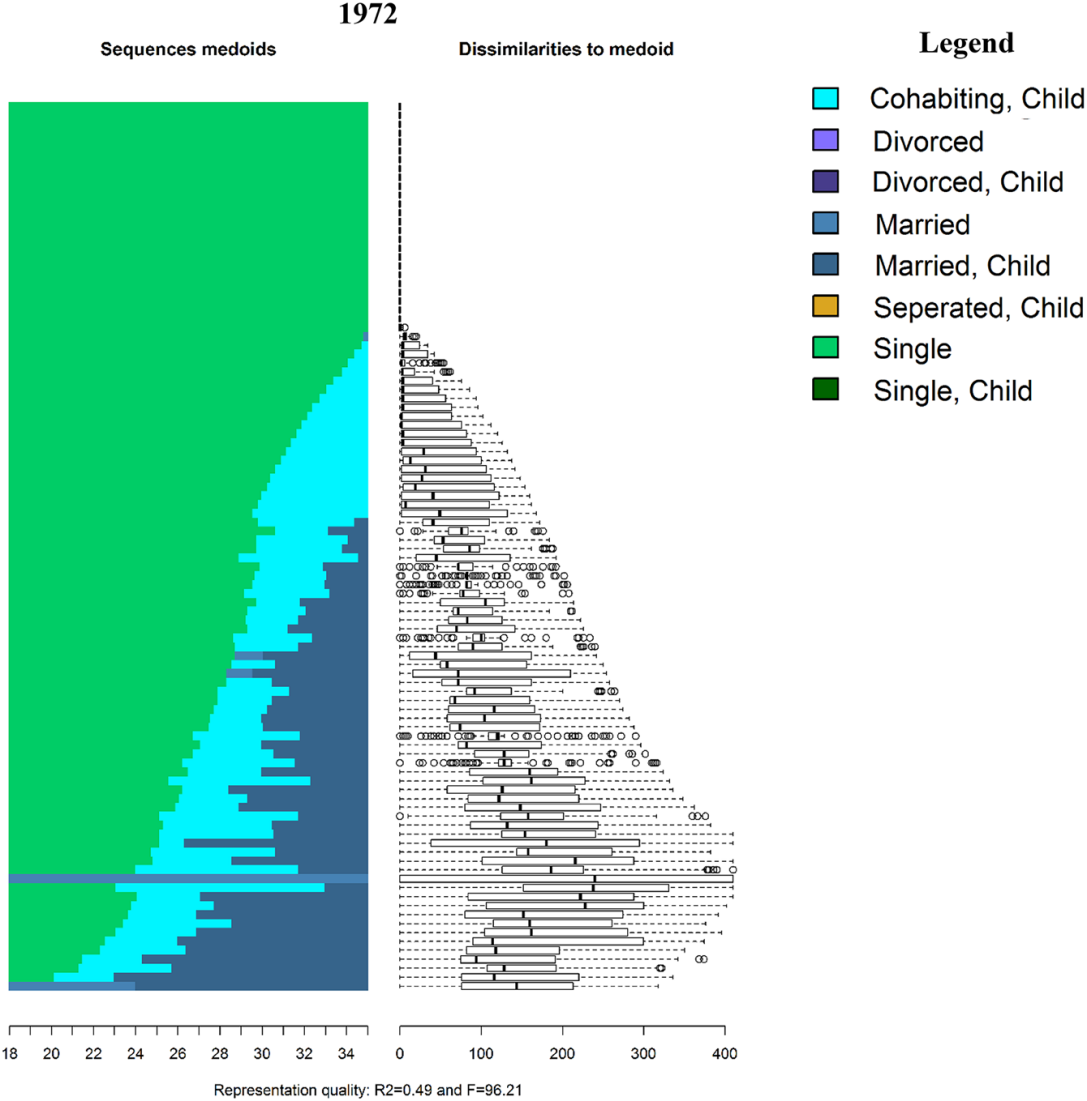
Relative frequency sequence plot of Swedish family formation trajectories by birth cohort

The family sequences of both Swedish men and women born in 1952, 1962 and 1972 from age 18 to 35 are displayed in Fig. 1 as relative frequency sequence plots (see Fasang and Liao 2014).^4^ Sequence index plots display all trajectories as they unfold horizontally stacked on top of each other vertically. In other words, the sequence number, i.e. observation 1, 2, 3, etc., is shown on the *y*-axis and time, i.e. age, on the *x*-axis. A disadvantage of sequence index plots is “over plotting”: individual sequences are no longer discernible due the large number of sequences being plotted. I therefore use relative frequency sequence plots to display a representative subset of sequences. Relative frequency plots are generated in five steps: (1) sequences are sorted by a chosen criterion, (2) the sorted sample is divided into subgroups, (3) a medoid sequence, i.e. the most representative sequence, is extracted from each subgroup, (4) the medoid sequences are plotted as index plots, and (5) the dissimilarity of sequences to the medoid within each subgroup are displayed as boxplots next the medoids. The final step indicates how well the medoids represent their subgroup. Further, *R*^2^ and *F* statistics are calculated to evaluate the overall goodness of fit for the chosen set of medoid sequences. I use average normalized distance as a sorting criterion and divide the sample into 100 subsamples to extract 100 medoid sequences.

The subset of medoid sequences for each cohort can be seen on the left panel and their dissimilarities on the right. For example, the sequence at the top of the index plot for the 1952 cohort consists of marriage without children (light blue) from age 18 until roughly age 22, followed by marriage with children (dark blue). On the right, the first boxplot indicates that the medoid is a good representative for its subsample, because median dissimilarity and the spread of the distribution are low. The top medoid for the 1962 cohort in contrast consists of a long spell of singlehood without children (light green) followed by cohabitation with children (turquoise) starting approximately at age 22.

Two differences can be seen in Fig. 1. First, there has been a dramatic delay in active family formation across birth cohorts. Not only has the average age at which individuals experience their first transition increased, but the proportion of individuals that neither marry nor enter parenthood before age 35 has increased. The median age of first marriage increased from 27.8 for the 1952 cohort to 30.5 for individuals born in 1972.^5^ Similarly, the median age of first birth increased from 25.5 and 28.5 between the 1952 and 1972 cohorts. Further, although nearly 60% of the 1952 cohort married by age 35, only 38% of the 1972 cohort married by age 35. The per cent of individuals that entered parenthood before age 35 dropped from 73% for the 1952 cohort to 69% of the 1972 cohort.

Second, there has been a shift in the first family state in the life course. Among those that began active family formation before age 35, most members of the 1952 cohort entered marriage before parenthood, while most members of the 1972 cohort entered parenthood within nonmarital cohabitation. The prevalence of divorce before age 35 decreased from 9% of the 1952 cohort to only 4% of the 1972 cohort, likely because of the delay and replacement of marriage with cohabitation. Therefore, most individuals that experience separations or divorce do so after age 35. For example, over 50% of the 1962 cohort that experience a divorce did so after the age of 35.^6^

These differences are reflected in how the most and least similar family patterns changed across cohorts. The family sequences in Fig. 1 are sorted from most to least dissimilar. Three changes can be observed across birth cohorts. First, while the most similar life course pattern in the 1952 cohort was marriage at age 18 followed by parenthood, the most similar life course patterns for the 1962 and 1972 birth cohorts consist of a delay in active family formation. Second, the most dissimilar life courses shifted in the same manner: continuous singlehood or late parenthood within cohabitation was most dissimilar for the 1952 cohort and early family formation became the most dissimilar pattern for the 1962 and 1972 cohorts. Finally, the moderately similar or dissimilar patterns, i.e. the medoid sequences between the most and least dissimilar, changed in both timing and sequencing across cohorts. Delayed marriage followed by parenthood was moderately dissimilar for both the 1952 and 1962 cohorts, but short spells of parenthood within cohabitation preceding marriage are increasingly common among the 1962 cohort. For the 1972 cohort, nearly all moderately dissimilar sequences consist of late entry into parenthood within cohabitation followed by marriage a few years later.

### Compositional Differences between Birth Cohorts

Summary statistics are displayed in Table 3 by birth cohort and gender. Average sequence dissimilarity is 43.9 for men born in 1952, which indicates that on average the edits cost 43.9 to transform one man’s sequence into another sequence within the 1952 birth cohort. However, the average dissimilarity of men’s family sequences dropped to 41.2 for the 1962 cohort and to 33.2 for the 1972 cohort. This indicates that men’s early family life courses have become more similar in Sweden across birth cohorts. Although women’s family sequences are on average less similar than men’s, average dissimilarity for women also decreased from 44.8 for the 1952 cohort to 37.3 for the 1972 cohort. This is clear evidence for hypothesis H2 that early family life courses have become more standardized across birth cohorts.

**Table 3:** Summary statistics by birth cohort

	1952	1962	1972
Distance			
Men	43.92	41.22	33.25
Women	44.82	42.71	37.37
Family structure single parent			
Men	0.16	0.24	0.29
Women	0.16	0.25	0.30
Mother’s age at 1st birth			
Men	24.63	23.45	23.38
Women	24.46	23.49	23.38
Parental education (ref. upper-secondary)			
No lower-secondary			
Men	0.47	0.28	0.08
Women	0.49	0.27	0.09
Lower-secondary			
Men	0.06	0.06	0.06
Women	0.06	0.05	0.06
Secondary			
Men	0.34	0.44	0.52
Women	0.34	0.45	0.51
Post-secondary			
Men	0.13	0.22	0.35
Women	0.11	0.23	0.34
Parental income (in 100,000 SEK)			
Men	3.16	3.99	4.64
Women	3.07	4.02	4.64
Educational attainment (ref. upper-secondary)			
Lower-secondary			
Men	0.26	0.17	0.10
Women	0.22	0.12	0.06
Secondary			
Men	0.47	0.56	0.52
Women	0.50	0.47	0.46
Upper-secondary			
Men	0.27	0.27	0.38
Women	0.28	0.31	0.48
Work experience			
Men	16.31	17.14	16.33
Women	15.24	17.00	16.20

As expected, educational attainment, parental education and income, as well as the proportion of individuals that lived in a single-parent household during childhood increased across birth cohorts. Average parental income at age 16 for women born in 1952 was 307,000 SEK, which increased to 402,000 SEK for the 1962 cohort and 464,000 SEK for the 1972 cohort. Similarly, the proportion of women from households where parents had less than 9 years of schooling dropped from nearly 50% of the 1952 cohort to 9% of the 1972 cohort. The proportion of women that lived in a single-parent household before age 16 increased from 16% for the 1952 cohort to 30% for the 1972 cohort. As could be expected, there are no substantial differences between gender regarding parental resources and childhood family structure.

However, there are gender differences regarding educational attainment and labour market experience. Although men and women born in 1952 exhibited similar levels of educational attainment, the proportion of women born in 1972 with more than upper-secondary education is 10 percentage points higher compared to men. Both men and women have high levels of work experience: men born in 1952 worked an average of 16.3 years between ages 18 and 35, while women worked an average of 15.2 years. And while the number of years worked increased between the 1952 and 1962 cohorts, both men and women born in 1972 worked fewer years than those born in 1962. Nonetheless, the gender difference dissipated for the 1972 cohort, where both men and women worked slightly more than 16 years on average.

### Decomposing Dissimilarity Differences Between Birth Cohorts

The results of Oaxaca–Blinder decompositions on the dissimilarity differentials between 1952–1962, 1952–1972, and 1962–1972 are displayed in Table 4 by gender. The average dissimilarity difference between cohorts is shown in the first row, followed by the compositional component and the associational component in the second and third rows, respectively. The components denote the cohort differences attributable to compositional and associational change. The proportion of the cohort difference that can be accounted for by compositional differences is displayed as a percentage in the fourth row. The detailed section shows cohort differences attributable to compositional shifts in specific indicators, e.g. parental education or work experience. Note that because the models are based on differences in dissimilarity, negative estimates denote a de-standardization and positive estimates indicate a standardization of early family sequences.^7^

**Table 4:** Oaxaca–Blinder decompositions on birth cohort differentials in average sequence dissimilarity

	Men			Women		
	1952–1962	1952–1972	1962–1972	1952–1962	1952–1972	1962–1972
Difference	2.70*** (0.15)	10.66*** (0.17)	7.97*** (0.16)	2.12*** (0.17)	7.45*** (0.20)	5.33*** (0.18)
Composition	− 0.10 (0.07)	0.30** (0.11)	0.37*** (0.07)	0.35*** (0.10)	1.15*** (0.14)	0.71*** (0.09)
Association	2.80*** (0.16)	10.37*** (0.20)	7.60*** (0.18)	1.77*** (0.18)	6.30*** (0.24)	4.62*** (0.17)
Per cent explained	− 3.70	2.81	4.64	16.50	15.43	13.32
*Detailed composition component*						
Family structure	− 0.09*** (0.02)	− 0.11*** (0.03)	− 0.04*** (0.01)	− 0.14*** (0.02)	− 0.16*** (0.03)	− 0.05*** (0.01)
Mother age at 1st birth	− 0.05* (0.02)	− 0.15*** (0.03)	− 0.01 (0.02)	− 0.13*** (0.02)	− 0.26*** (0.03)	− 0.03 (0.02)
Parental education	− 0.02 (0.04)	0.06 (0.09)	0.00 (0.05)	0.08 (0.04)	0.17 (0.10)	0.09* (0.05)
Parental income	0.03 (0.04)	0.18* (0.07)	0.05 (0.03)	0.20*** (0.04)	0.45*** (0.09)	0.18*** (0.03)
Educational attainment	0.06** (0.02)	0.32*** (0.05)	0.22*** (0.03)	0.24*** (0.04)	0.78*** (0.07)	0.63*** (0.05)
Work experience	− 0.04 (0.03)	− 0.00 (0.01)	0.15*** (0.03)	0.10 (0.06)	0.15*** (0.04)	− 0.11** (0.04)
*N*	8676	9082	9802	8690	8697	9383

Statistical significance: *p < 0.05; **p < 0.01; ***p < 0.001; Standard errors in parentheses

As discussed above and in line with hypothesis 2, average dissimilarity has decreased across cohort comparisons for both men and women. For example, between the 1952 and 1962 birth cohorts, average dissimilarity decreased by 2.7 for men and 2.1 for women. For men, none of the decrease in average dissimilarity can be accounted for by compositional differences. However, for women, 16.5% of the 1952–1962 cohort difference is attributable to compositional differences between cohorts. The compositional component indicates that 0.3 of the 2.1 edit cost difference is due to compositional shifts. In other words, had there been no compositional differences between the 1952 and 1962 birth cohorts, the cohort difference would have been 1.7 rather than 2.1. For women, the results are similar for the other cohort comparisons: 15.4% of 1952–1972 and 13.3% of the 1962–1972 dissimilarity gaps can be accounted for by compositional change. For men, the proportion is much smaller: only 2.8 and 4.6% of the 1952–1972 and 1962–1972 difference, respectively, is attributable to compositional shifts.

Compositional differences in parental resources are statistically associated with decreasing dissimilarity for women, but less so for men. For example, the compositional component for parental income indicates that 0.2 of the 2.1 edit cost difference between the 1952 and 1962 cohorts can be accounted for by an increase in parental income. Similarly, increasing parental income accounts for 6% of the 1952–1972 decrease in dissimilarity and 3.3% of the 1962–1972 decrease. Percentages are calculated by dividing the detailed composition component by the total differences and multiplying by 100, e.g. (0.2/2.1) × 100 for parental income and women’s 1952–1972 dissimilarity gap. For men, increasing parental income is only statistically associated with the change in dissimilarity between the 1952 and 1972 cohorts. There are no substantial associations between increasing parental education and decreasing dissimilarity. Only for the 1962–1972 comparison does parental education have a small but statistically significant positive influence on women’s change in average dissimilarity. In sum, my results support hypothesis H2A for women: increasing parental income is associated with more standardized family trajectories.

Increases in individual’s own educational attainment are systematically associated with decreasing dissimilarity across cohorts. For men, 0.06 of the 2.7 dissimilarity differences between 1952 and 1962 can be accounted for by increasing educational attainment. Although similarly small, 3% of the 1952–1972 differences and 2% of the 1962–1972 difference are attributable to an increase in education. The associations are much more substantial for women: higher educational attainment accounts for between 10 and 11% of the decrease in average dissimilarity across all cohort comparisons. Therefore, there is clear support for my hypothesis H2B that an increase in average educational attainment will be associated with more standardized trajectories.

The empirical results related to labour market attachment are somewhat ambiguous. For three of the six cohort comparisons, changes in work experience during early adulthood are neither associated with an increase or a decrease in dissimilarity. For women, the increase in the number of years employed between 1952 and 1972 is associated with a 0.15 decrease in average dissimilarity. The subsequent decrease between 1962 and 1972 is associated with higher average distances. In contrast, the same decrease between 1962 and 1972 for men is associated with a decrease in dissimilarity. In sum, there is some support for hypothesis H2C that increases in work experience are associated with more standardized family life courses, however only for women.

Differences in childhood family structure across birth cohorts are, like educational attainment, systematically associated with change in average distance. However, rather than a shift towards more similar trajectories, increases in single-parent families are associated with more dissimilar sequences. For all cohort comparisons, average dissimilarity would have decreased to an even greater extent if there had been no increase in single-parent families during childhood. For example, the dissimilarity difference between the 1952 and 1962 cohorts for women would have increased by 0.14 from 2.12 to 2.26 for women. Therefore, my results indicate, in line with hypothesis H1D, that higher levels of single-parent childhood households are associated with less standardized family life courses.

As indicated in the discussion above, compositional shifts across cohorts have had a greater impact on change in the dissimilarity of women’s trajectories compared to men’s. The overall as well as the detailed composition components relative to the cohort differentials are generally larger for women compared to men. For example, change in parental income between the 1952 and 1972 cohorts accounts for 6% of the dissimilarity difference for women, but only 1.6% for men. Similarly, just 3% of men’s dissimilarity difference between the 1952 and 1972 cohorts is attributable to increasing educational attainment, but over 10% for women. These examples are clearly in line with hypothesis 3 that the associations between compositional differences and more standardized family life courses are stronger for women.

### Robustness Checks

I performed sensitivity analyses to address four main limitations of this study. First, I cannot use the residence registers to identify leaving the parental home or childless cohabitation. It is difficult to identify the date that children left the parental home, because children are often registered as residing with their parents while studying. Further, it is problematic to identify childless cohabitation, because children need to be used as proxies to identify cohabiting couples. The property number is the lowest-level geographical unit in the data, which may identify a single household or an apartment complex. I therefore assume that two individuals are cohabiting if they are registered at the same property number and have a child. Although there is some room for error, Thomson and Eriksson (2013) demonstrated that register estimates of cohabitation are consistent to census and survey based estimates. I preformed robustness checks with data from the Swedish Generations and Gender Survey (GGS) implementing information on parental home leaving and childless cohabitation in the family sequences (see section V in the online supplement). Further, these analyses use a broader 10-year cohort range, which additionally ensures that my analyses are not biased by using a single birth year. The results are similar to those presented above and lead to the same substantive conclusions.

Second, I attempted to increase the comparability of cohorts by calculating distances on 5-year sequences surrounding the average age of the first transition within each: 25–30 for the 1952 cohort, 27–32 for the 1972 cohort, and 30–35 for the 1972 cohort (see section VI in the online supplement). The results of these robustness checks underscore that compositional differences in educational attainment and parental income are associated with decreases in family life course dissimilarity, while change in childhood family structure increases dissimilarity. However, these analyses show no change or even an increase in dissimilarity across cohorts. This reflects the distribution of life course states during that timeframe: married with children is most common for the 1952 cohort, married with children as well as small portions of cohabitation with children and singlehood for the 1962 cohorts, and similar proportions of married with children, cohabitation with children and singlehood for the 1972 cohort. While it is important to study variability during these short transitional periods, it is equally important to study early family life courses during early adulthood when individuals are transitioning out of the educational system and into the labour market.

Third, I can only observe family life courses up to age 35 for the 1972 birth cohort. My results could be biased if dissimilar life course patterns manifest themselves only later in the life course. I generated family sequences from age 18 to 45 for men and women born in 1952 and in 1962 and replicated my analyses (see section I and III in the online supplement). Finally, I performed several robustness checks using different sequence state definitions (see sections II and III in the online supplement). For example, I replicated the analyses using sequence states where union statuses are differentiated by parity, (e.g. S, S1C, S2C, or S3C), sequences that are only differentiated by union status, e.g. S, M, C, Sp, D, as well as sequences that are only differentiated by parity, e.g. 1C, 2C, 3C, etc. The results of these robustness checks are overall very similar with those presented above.

## Discussion

In this study, I investigated (1) whether early family life courses have become more or less standardized across birth cohorts in Sweden and (2) to what extent increases or decreases in family life course standardization can be attributed to compositional changes. My results using Swedish register data and metrics developed in sequence analysis with Oaxaca–Blinder decompositions demonstrate that family formation has become more standardized across birth cohorts in Sweden (H2). For both men and women, higher-level educational attainment is associated with higher-level early family life course standardization (H2B). Further, the family life courses of men and women would have standardized to an even greater degree if experiencing single-parent families during childhood had not become more common (H1D). However, higher levels of parental resources, specifically income, are only associated with higher levels of early family life course standardization for women (H2A). Similarly, increasing work experience is only associated with increasing similarity for women (H2C). As expected, compositional changes are more strongly associated with women’s early family life course standardization (H3).

My study adds at least four empirical and subsequently theoretical insights to the literature on family life course change. First, I demonstrate, that in contrast to common conceptions, early family life courses have become more standardized. This finding adds evidence to recent studies that have challenged the notion of an irreversible decrease in life course standardization into question (e.g. Zimmermann and Konietzka 2017). My results substantiate Huinink’s (2013) theoretical argument that family formation may re-standardized if change is a transitory process. Indeed, the standardization of early family life courses is the result of two processes: (1) a dramatic delay in active family formation and (2) a shift from parenthood within marriage to parenthood within cohabitation followed by marriage. This demonstrates that it is important to study family formation as a process outcome (Abbott 2005), because standardization resulted from longer durations outside marriage and parenthood as well as a shift in the ordering of life course events.

Focusing on holistic family trajectories and conceptualizing change in terms of a transitional process has important implications for the empirical and conceptual validity of the SDT thesis. Although many of the current trends in family demographic behaviour correspond with the SDT, especially higher rates of cohabitation and the postponement of marriage and parenthood (Cherlin 2012; Ortega 2014; Zeman et al. 2018), a number of trends match up with the SDT to a lesser degree, such as the relative stabilization of marriage, divorce, and fertility rates in recent decades (Schoen and Standish 2001; Andersson et al. 2009; Ohlsson-Wijk 2011). However, my study demonstrates that irreversible change in single elements of family demographic behaviour does not imply an irreversible de-standardization of family formation. In contrast, change in the prevalence, timing, and ordering of cohabitation, marriage, and parenthood established a new standardized pattern of early family formation in Sweden. A reformulation of the SDT thesis should entail identifying which elements of family demographic changes are persistent and how they might translate into new dominant patterns of family formation.

Second, I show that compositional shifts in individual characteristics are indeed associated with change in family life courses. My results indicate that a population-level increase in educational attainment across birth cohorts is the main compositional driver of the standardization of early family life courses for men and women. The opportunity costs of parenthood and marriage are higher for a more educated population, which results in delayed entry into parenthood and a greater prevalence of cohabitation (Becker 1974b; Becker et al. 1977; Becker and Tomes 1994). A more educated population also holds more gender-egalitarian norms, which stabilizes fertility rates (Esping-Andersen and Billari 2015; Goldscheider et al. 2015). In sum, the expansion of the education system in Sweden facilitated a shift from a pattern of early marriage and parenthood to delayed parenthood with cohabitation by increasing opportunity costs and promoting gender egalitarianism.

Third, my results highlight gender differences in the relationship between compositional shifts and the standardization of early family life courses. My results show that compositional shifts in educational attainment account for the standardization of family life courses to a greater degree for women than for men. This reflects the reversal of the gender gap in educational attainment. While both men and women profited from the expansion of the educational system, women are now more educated than men in Sweden. Moreover, compositional shifts in parental incomes are associated with more standardized life courses for women, but not for men. Therefore, the processes that linked higher educational attainment and more standardized family formation for women were reinforced by higher standards of living during childhood. Especially during times of increased economic uncertainty, women may delay marriage and parenthood until they have secured a standard of living that matches what they grew accustomed to during childhood (Easterlin 1975, 1976; Oppenheimer 1988).

Fourth, my analyses demonstrate that compositional differences between cohorts may work in an opposite manner. The best example for this is how change in childhood family structure is associated with family life course change. Rather than reinforcing the trend towards more standardized early life courses, the increase in single-parent families across cohorts dampened this trend. Specifically, I show that early family trajectories would have become even more similar had the proportion of single-parent families not increased.

The main methodological contribution to the literature is the introduction of an alternative approach to hazard models and standardization techniques (e.g. Neels et al. 2017 on educational participation and later childbearing) to quantify compositional change and link individual characteristics to population change. This methodological approach enables family sociologists and demographers to quantify the proportion of population change that is attributable to overall compositional change and detailed decompositions allow researchers to establish a link between single micro-level characteristics and population change. This is especially useful when testing theoretically derived hypotheses. Combining sequence analysis with decomposition models is applicable to other areas of life course research that seek to understand cross-temporal or cross-nation variation in life courses at the population level, such as the fractionalization of employment careers (e.g. Van Winkle and Fasang 2017).

One limitation of this study remains that can only observe my youngest cohort from age 18 to 35. Although scholars contended that age 16–35 is the most demographically dense phase in individuals’ life courses (Cook and Furstenberg 2002), it seems that this window has grown wider. My robustness checks with longer sequences from age 18 to 45 for the 1952 and 1962 bolster my findings, but it is an empirical question whether the same holds for younger cohorts. Nonetheless, I contend that for men and women born in 1972, a long period without family commitments seems to be a near universal experience during the transition to adulthood. Further research should investigate trends in family life course standardization past early adulthood. A further limitation surrounds the causal link between changes in observed characteristics and family life course standardization. It is likely that compositional changes in educational attainment, for example, and family life courses are tightly intertwined and mutually reinforce each other. Further, my analyses rely on associations, not effects. Therefore, I am not able to make any causal claims.

In this study, I demonstrated that compositional changes can account for some change in family life courses. However, over four-fifths of the decrease in early life course dissimilarity cannot be accounted for by compositional shifts in the characteristics I observe. Across all comparisons, the decrease in dissimilarity is largely accounted for by associational differences, i.e. different associations between my observed characteristics and dissimilarity. For example, the association between parental income and dissimilarity has increased across cohort, which has also driven the trend towards standardization. These could result from external differences between cohorts. For example, parental income may be more important for younger cohorts that experience more uncertainty during their transitions to adulthood within the wider context of globalization. Moreover, change in Swedish family policy and family values may induce behavioural differences not easily captured by compositional change in the characteristics I could observe. For example, a shift individuals’ conceptions of the ideal age of marriage is likely a driver in the standardization of early family life courses.

Future cross-national research should uncover whether a standardization of early family life courses can be observed in other contexts as well. For example, have early family life courses also become more standardized in the USA? Similar to Sweden, the expansion of the educational system may also incite a delay in active family formation. However, educational attainment is more stratified by social background, which may increase the importance of childhood family structure. More generally, my results indicate that the experience of early family formation has become more equal in Sweden. Future research should investigate what this equality of experiences means subjectively and what consequences it has for individuals and society.

### Electronic supplementary material

Below is the link to the electronic supplementary material.

Supplementary file1 (PDF 914 kb)

## Appendix

See Tables 5 and 6.

**Table 5:** OLS regression results on average sequence dissimilarity by birth cohort

	Men			Women		
	1952	1962	1972	1952	1962	1972
Family structure						
Single parent	1.28*** (0.29)	0.94*** (0.28)	0.51^+^ (0.29)	1.55*** (0.31)	1.45*** (0.27)	0.68* (0.34)
Mother’s age at 1st birth	0.00 (0.02)	− 0.08** (0.03)	− 0.26*** (0.03)	− 0.10*** (0.02)	− 0.17*** (0.03)	− 0.41*** (0.04)
Parental education (ref. upper-secondary)						
No lower-secondary	0.05 (0.24)	− 0.24 (0.26)	0.46 (0.53)	0.06 (0.24)	0.39 (0.28)	0.43 (0.56)
Lower-secondary	− 0.44 (0.48)	0.30 (0.57)	0.39 (0.59)	− 0.18 (0.53)	− 0.03 (0.47)	− 0.82 (0.65)
Post-secondary	0.31 (0.39)	− 0.24 (0.32)	− 0.32 (0.26)	− 0.55 (0.40)	0.00 (0.33)	− 0.81* (0.37)
Parental income	− 0.11 (0.07)	0.04 (0.08)	− 0.16* (0.07)	− 0.22** (0.07)	− 0.21** (0.08)	− 0.34*** (0.08)
Educational attainment (ref. upper-secondary)						
Lower-secondary	0.55* (0.25)	0.76* (0.30)	1.83*** (0.54)	1.50*** (0.32)	2.65*** (0.39)	3.23*** (0.72)
Post-secondary	− 1.42*** (0.27)	− 0.62* (0.30)	− 1.86*** (0.26)	− 1.56*** (0.26)	− 2.20*** (0.24)	− 3.34*** (0.32)
Work experience	− 0.01 (0.04)	0.09^+^ (0.05)	0.23*** (0.04)	− 0.07^+^ (0.04)	− 0.03 (0.06)	− 0.15* (0.06)
Constant	44.28*** (1.03)	41.40*** (1.17)	36.74*** (1.17)	48.85*** (0.91)	47.79*** (1.29)	52.47*** (1.38)
Adj. *R*^2^	0.019	0.010	0.054	0.052	0.077	0.109
*N*	3978	4698	5104	4002	4688	4695

Statistical significance: **p* < 0.05; ***p* < 0.01; ****p* < 0.001; Standard errors in parentheses

**Table 6:** Oaxaca–Blinder decompositions on birth cohort differentials in average sequence dissimilarity

	Men			Women		
	1952–1962	1952–1972	1962–1972	1952–1962	1952–1972	1962–1972
Difference	2.70*** (0.15)	10.66*** (0.17)	7.97*** (0.16)	2.12*** (0.17)	7.45*** (0.20)	5.33*** (0.18)
Composition	− 0.10 (0.07)	0.30** (0.11)	0.37*** (0.07)	0.35*** (0.10)	1.15*** (0.14)	0.71*** (0.09)
Association	2.80*** (0.16)	10.37*** (0.20)	7.60*** (0.18)	1.77*** (0.18)	6.30*** (0.24)	4.62*** (0.17)
Per cent explained	− 3.70	2.81	4.64	16.50	15.43	13.32
*Detailed association component*						
Family structure	0.07 (0.08)	0.16 (0.08)	0.11 (0.10)	0.03 (0.08)	0.20* (0.10)	0.21 (0.12)
Mother age at 1st birth	2.07* (0.84)	6.43*** (0.93)	4.27*** (0.87)	1.57* (0.76)	7.48*** (1.14)	5.81*** (1.15)
Parental education	0.16 (0.21)	0.02 (0.18)	− 0.06 (0.16)	− 0.23 (0.20)	0.06 (0.20)	0.30 (0.19)
Parental income	− 0.55 (0.37)	0.19 (0.39)	0.85* (0.39)	− 0.03 (0.37)	0.45 (0.44)	0.55 (0.44)
Educational attainment	− 0.26 (0.14)	− 0.04 (0.16)	0.26 (0.17)	0.01 (0.16)	0.49* (0.20)	0.40* (0.18)
Work experience	− 1.56 (1.04)	− 3.95*** (1.07)	− 2.49* (1.15)	− 0.63 (1.12)	1.23 (1.29)	2.02 (1.50)
Constant	2.88 (1.52)	7.54*** (1.66)	4.66** (1.64)	1.05 (1.48)	− 3.62 (1.89)	− 4.67* (1.99)
*N*	8676	9082	9802	8690	8697	9383

Statistical significance: **p* < 0.05; ***p* < 0.01; ****p* < 0.001; Standard errors in parentheses

## Footnotes

1 Incomes are inflation adjusted to 2017 SEK.

2 I use the Sweden in Time: Activities and Relationships (STAR) data, a collection of variables from population registers and censuses produced by Statistics Sweden. Anyone that was registered in Sweden in 1968 or later or in at least one of the censuses 1960–1990 is included, which amounts to the entire Swedish population. The register variables are made available through remote access to projects developed at the Institute for Social Research (SOFI) at Stockholm University and the Stockholm University Demography Unit (SUDA).

3 Sequence analysis is performed using the TraMineR package in R (Gabadinho et al. 2011).

4 The dissimilarities are calculated using OM distance. The plots were created with the *seqplot.rf* function developed by Matthias Studer, Anette Fasang, and Tim Liao implemented in the *TraMineRextras* package using R, version 3.2.0.

5 Note these are lower bound estimates of the median age of first marriage and birth for the 1972 birth cohort, because events after the age of 35 have not yet been observed.

6 Note these are lower bound estimates of the median age of first marriage and birth for the 1962 birth cohort, because events after the age of 45 have not yet been observed.

7 For example, if average distance is 44 for the 1952 cohort and 41 for the 1962 cohort, then the average difference in dissimilarity is 3 (44–41).
