# Supplementary material for: Early Family Life Course Standardization in Sweden: The Role of Compositional Change
Source: Eur J Popul. 2020 Jan 13;36(4):765–98. doi: 10.1007/s10680-019-09551-y (PMC7492312; doi:10.1007/s10680-019-09551-y)
Supplement: Supplementary file 1 — Supplementary file1 (PDF 914 kb) [file 10680_2019_9551_MOESM1_ESM.pdf]

Family Life Course Standardization in Sweden: the Role of Compositional Change

Online Supplement

Section I: Long Sequences with Simple State Alphabet

Figure 1: Relative Frequency Sequence Plot of Long Swedish Family Formation Trajectories by Birth Cohort

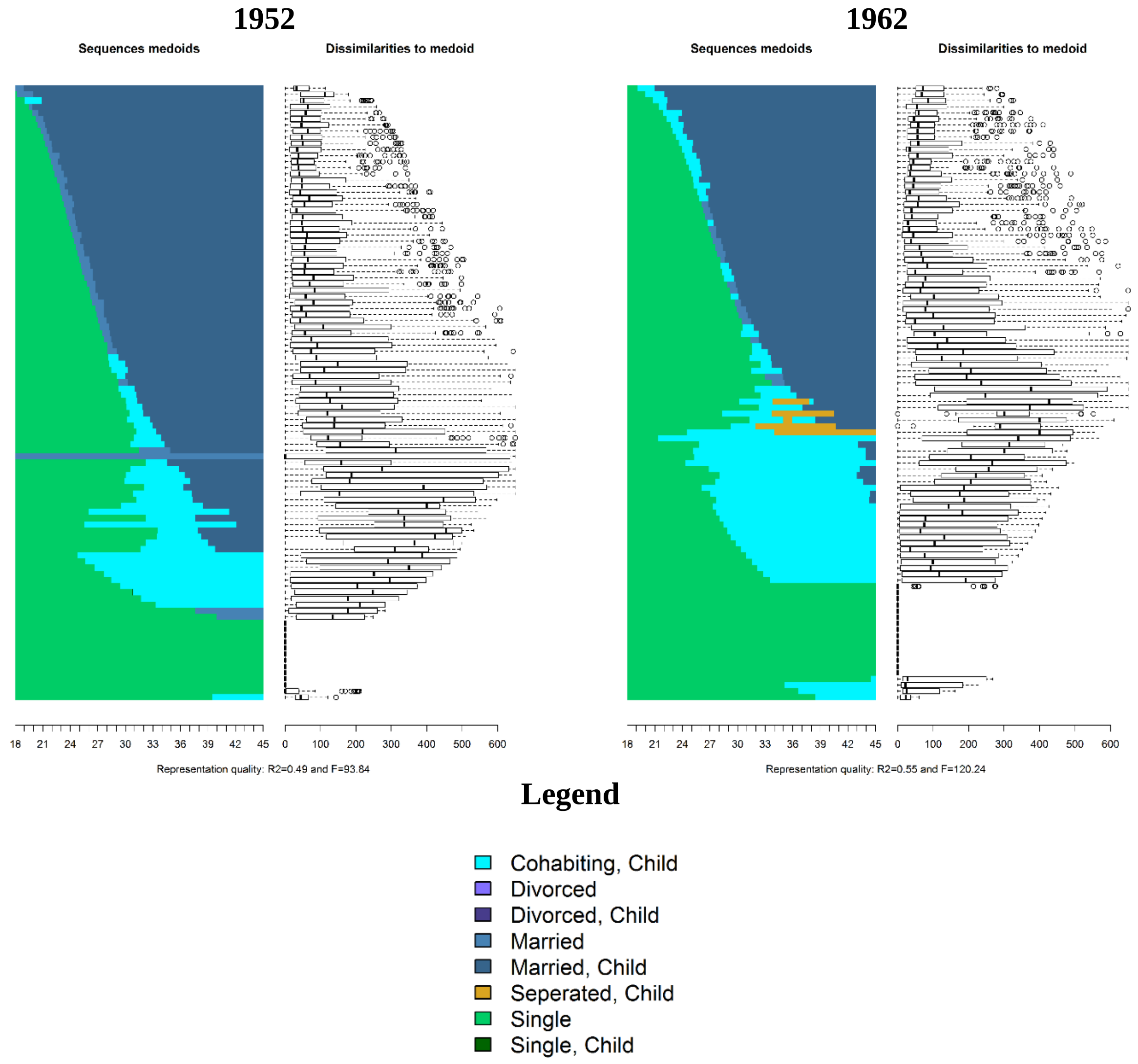

Table 1: Oaxaca-Blinder Decompositions on Birth Cohort Differentials in Average Sequence Dissimilarity – Long Swedish Sequences

	Men	Women
Average Distance		
1952	42.93***	43.98***
	(0.10)	(0.12)
1962	42.33***	44.04***
	(0.07)	(0.09)
Difference	0.60***	-0.06
	(0.12)	(0.15)
Composition	0.15**	0.37***
	(0.05)	(0.08)
Association	0.45***	-0.43**
	(0.13)	(0.16)
Percent Explained	25.00	N/A
Detailed Composition Components		
Family Structure	-0.08***	-0.15***
	(0.01)	(0.02)
Mother Age at	-0.06***	-0.14***
1st Birth	(0.01)	(0.02)
Parental Education	0.04	0.01
	(0.03)	(0.04)
Parental Income	0.04	0.19***
	(0.04)	(0.04)
Educational	0.06**	0.19***
Attainment	(0.02)	(0.03)
Work Experience	0.14***	0.27***
	(0.02)	(0.05)

Table 1 continued

Detailed Association Component		
Family Structure	0.12	-0.00
	(0.06)	(0.08)
Mother Age at 1st Birth	1.13	0.75
	(0.64)	(0.73)
Parental Education	0.19	0.21
	(0.17)	(0.19)
Parental Income	-0.13	-0.22
	(0.26)	(0.30)
Educational Attainment	-0.11	0.06
	(0.12)	(0.14)
Work Experience	-0.72	1.03
	(1.09)	(1.16)
Constant	-0.03	-2.26
	(1.35)	(1.54)
N	9258	8930

Note: Statistical significance: * $p < 0.05$, ** $p < 0.01$, *** $p < 0.001$; Standard errors in parentheses.

Section II: Sequences with State Alphabet Differentiated by Parity& Living Arrangements

Figure 1: Relative Frequency Sequence Plot of Swedish Family Formation Trajectories by Birth Cohort

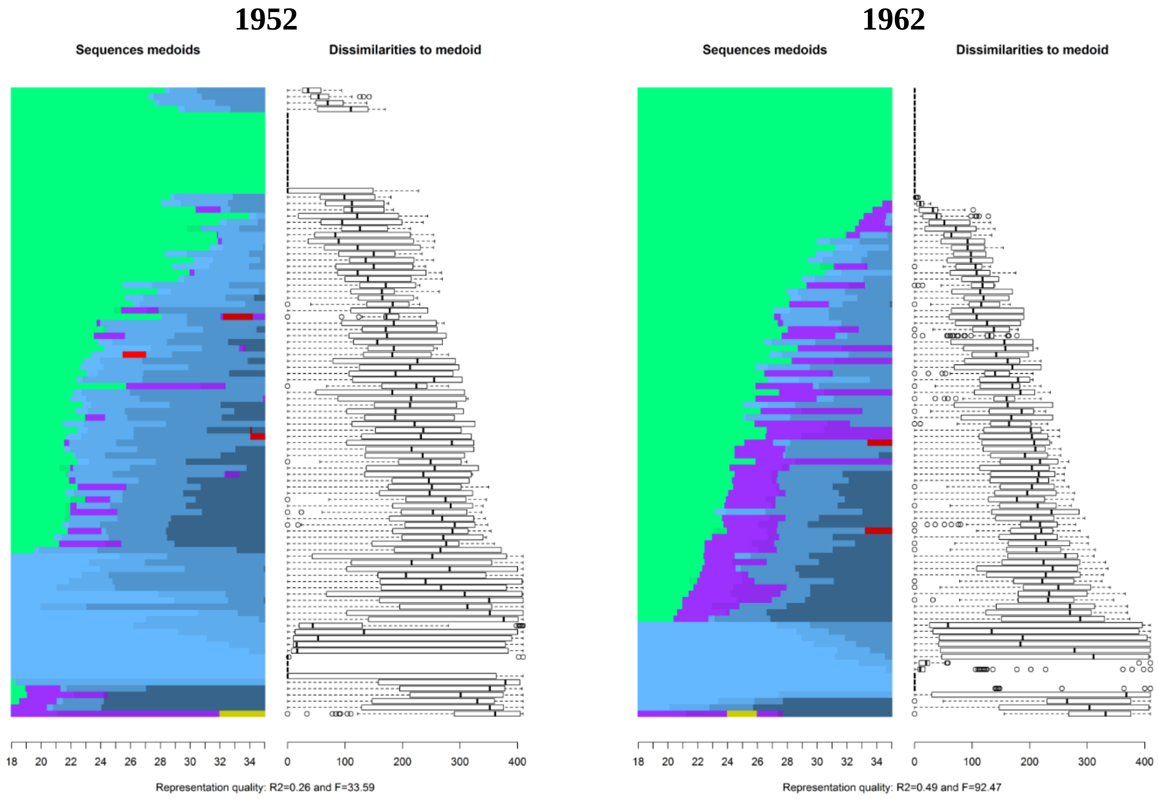

1972

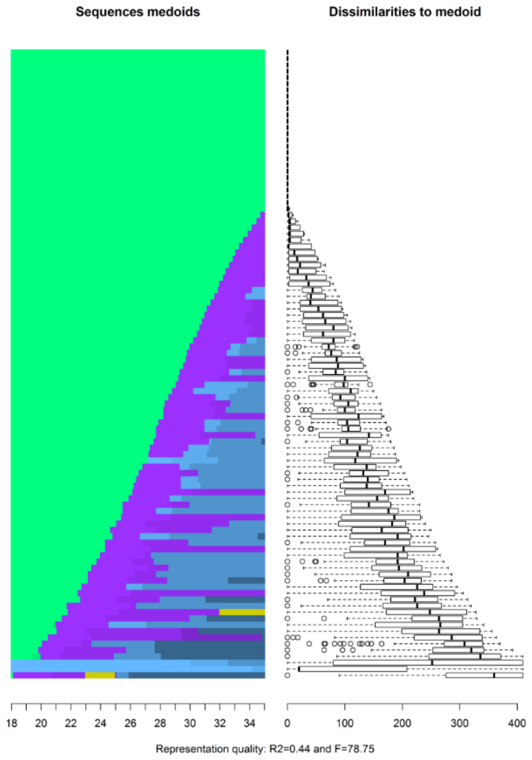

Sequences medoids
Dissimilarities to medoid
18 20 22 24 26 28 30 32 34
0 100 200 300 400
Representation quality: R2=0.44 and F=78.75

Legend

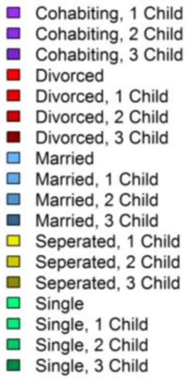

Cohabiting, 1 Child
Cohabiting, 2 Child
Cohabiting, 3 Child
Divorced
Divorced, 1 Child
Divorced, 2 Child
Divorced, 3 Child
Married
Married, 1 Child
Married, 2 Child
Married, 3 Child
Seperated, 1 Child
Seperated, 2 Child
Seperated, 3 Child
Single
Single, 1 Child
Single, 2 Child
Single, 3 Child

Table 1: Summary Statistics by Birth Cohort

		1952	1962	1972
Distance				
	Men	48.25	44.12	34.56
	Women	50.65	46.80	39.19
Family Structure				
Single Parent				
	Men	0.16	0.25	0.28
	Women	0.16	0.25	0.29
Mother's Age at 1st Birth				
	Men	24.59	23.50	23.44
	Women	24.57	23.40	23.38
Parental Education (ref. Upper-Secondary)				
No Lower-Secondary				
	Men	0.47	0.28	0.08
	Women	0.50	0.28	0.09
Lower-Secondary				
	Men	0.06	0.05	0.06
	Women	0.06	0.05	0.06
Secondary				
	Men	0.35	0.55	0.52
	Women	0.33	0.55	0.51
Post-Secondary				
	Men	0.12	0.22	0.34
	Women	0.11	0.22	0.34
Parental Income (in 100,000 SEK)				
	Men	3.13	3.98	4.64
	Women	3.10	3.97	4.63
Educational Attainment (ref. Upper-Secondary)				
Lower-Secondary				
	Men	0.27	0.17	0.09
	Women	0.21	0.13	0.06
Secondary				
	Men	0.46	0.55	0.53
	Women	0.48	0.57	0.45
Upper-Secondary				
	Men	0.27	0.28	0.38
	Women	0.31	0.30	0.49
Work Experience				
	Men	16.36	17.10	16.36
	Women	15.26	16.89	16.24

Table 2: OLS Regression Results on Average Sequence Dissimilarity by Birth Cohort

	Men			Women		
	1952	1962	1972	1952	1962	1972
Family Structure						
Single Parent	0.95***	1.00***	0.63*	0.92***	0.84**	0.76*
	(0.29)	(0.28)	(0.31)	(0.25)	(0.30)	(0.37)
Mother's Age	-0.08***	-0.17***	-0.21***	-0.17***	-0.25***	-0.44***
at 1st Birth	(0.02)	(0.03)	(0.04)	(0.02)	(0.03)	(0.04)
Parental Education						
(ref. Upper-Secondary)						
No Lower-Secondary	-0.03	-0.08	0.30	0.29	0.04	1.43*
	(0.21)	(0.29)	(0.56)	(0.23)	(0.30)	(0.62)
Lower-Secondary	-0.31	-0.15	0.72	0.38	0.17	0.31
	(0.37)	(0.53)	(0.63)	(0.47)	(0.54)	(0.76)
Post-Secondary	-0.43	-0.14	-0.39	-0.37	-0.57^{+}	-0.90*
	(0.35)	(0.35)	(0.30)	(0.33)	(0.32)	(0.39)
Parental Income	-0.04	0.08	-0.09	-0.34***	-0.10	-0.21*
	(0.06)	(0.08)	(0.08)	(0.05)	(0.08)	(0.09)
Educational Attainment						
(ref. Upper-Secondary)						
Lower-Secondary	0.22	0.86*	2.20***	1.34***	3.19***	1.25
	(0.23)	(0.36)	(0.58)	(0.26)	(0.46)	(0.90)
Post-Secondary	-0.43^{+}	-0.70*	-2.15***	-2.76***	-2.48***	-3.84***
	(0.24)	(0.28)	(0.29)	(0.22)	(0.29)	(0.32)
Work Experience	0.33***	0.29***	0.39***	-0.34***	0.01	-0.18*
	(0.05)	(0.06)	(0.05)	(0.04)	(0.07)	(0.08)
Constant	44.96***	42.62***	34.08***	61.37***	53.16***	55.17***
	(1.00)	(1.15)	(1.30)	(0.85)	(1.40)	(1.71)
Adj. R^2	0.031	0.021	0.050	0.136	0.085	0.098
N	4016	4661	5124	4066	4733	4696

Note: Statistical significance: * $p < 0.05$, ** $p < 0.01$, *** $p < 0.001$; Standard errors in parentheses.

Table 3: Oaxaca-Blinder Decompositions on Birth Cohort Differentials in Average Sequence Dissimilarity

	Men			Women		
	1952-1962	1952-1972	1962-1972	1952-1962	1952-1972	1962-1972
Difference	4.13***	13.70***	9.57***	3.85***	11.46***	7.61***
	(0.16)	(0.17)	(0.19)	(0.16)	(0.19)	(0.20)
Composition	-0.40***	0.21	0.49***	0.41***	1.41***	0.91***
	(0.07)	(0.12)	(0.07)	(0.11)	(0.14)	(0.10)
Association	4.53***	13.49***	9.07***	3.44***	10.05***	6.70***
	(0.16)	(0.20)	(0.21)	(0.18)	(0.23)	(0.21)
Percent Explained	-9.68	1.53	5.12	10.64	12.30	11.95
Detailed Composition Component						
Family Structure	-0.09***	-0.10***	-0.03*	-0.08***	-0.11**	-0.03*
	(0.02)	(0.03)	(0.01)	(0.02)	(0.04)	(0.01)
Mother Age at 1st Birth	-0.14***	-0.17***	-0.01	-0.25***	-0.34***	-0.01
	(0.02)	(0.02)	(0.02)	(0.03)	(0.04)	(0.03)
Parental Education	0.02	0.13	0.02	0.09*	0.44***	0.16**
	(0.04)	(0.10)	(0.05)	(0.04)	(0.12)	(0.06)
Parental Income	-0.02	0.08	0.01	0.18***	0.40***	0.10**
	(0.04)	(0.08)	(0.04)	(0.04)	(0.09)	(0.04)
Educational Attainment	0.06*	0.28***	0.25***	0.15***	0.77***	0.77***
	(0.02)	(0.05)	(0.03)	(0.04)	(0.07)	(0.06)
Work Experience	-0.23***	-0.00	0.26***	0.32***	0.27***	-0.08*
	(0.03)	(0.02)	(0.03)	(0.07)	(0.05)	(0.03)
N	8677	9140	9785	8799	8762	9429

Note: Statistical significance: * $p < 0.05$, ** $p < 0.01$, *** $p < 0.001$; Standard errors in parentheses.

Section III: Long Sequences with State Alphabet Differentiated by Parity & Living Arrangements

Figure 1: Relative Frequency Sequence Plot of Long Swedish Family Formation Trajectories by Birth Cohort

1952 **1962**

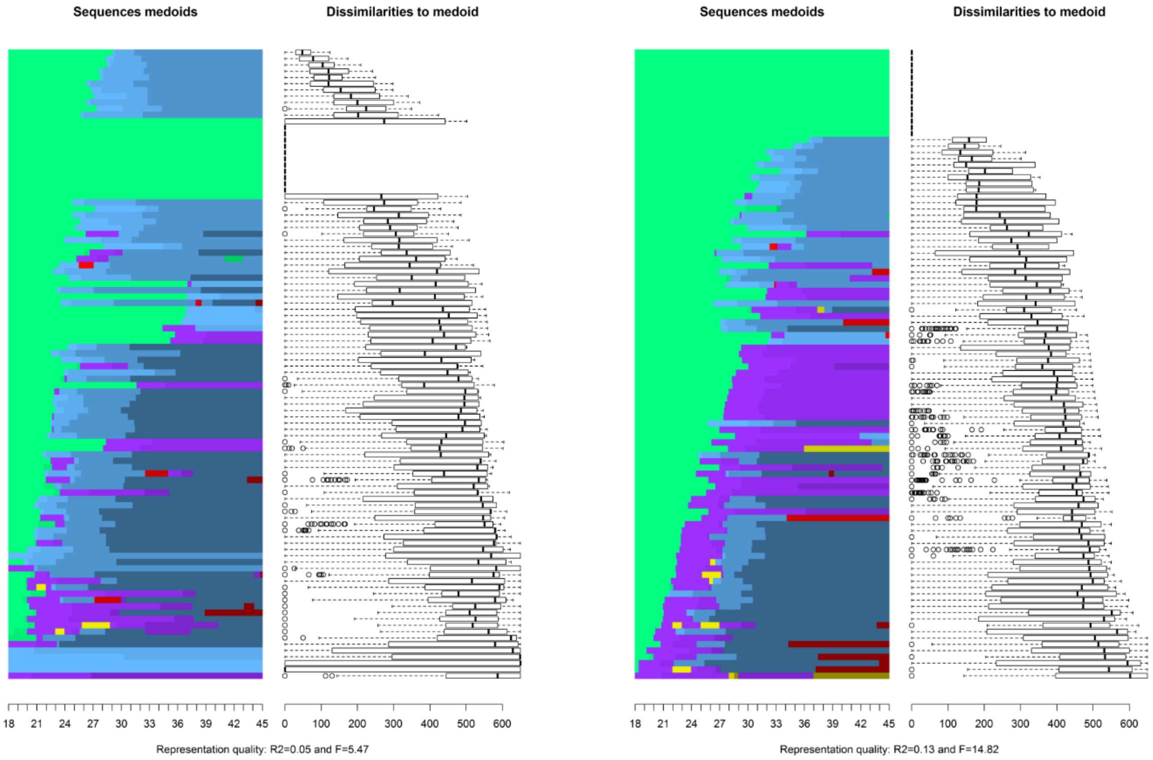

Legend

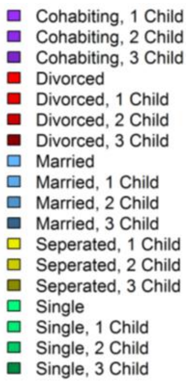

Table 1: Oaxaca-Blinder Decompositions on Birth Cohort Differentials in Average Sequence Dissimilarity – Long Swedish Sequences

	Men	Women
Average Distance		
1952	49.86^{***}	52.17^{***}
	(0.07)	(0.09)
1962	47.02^{***}	49.62^{***}
	(0.07)	(0.09)
Difference	2.84^{***}	2.54^{***}
	(0.10)	(0.13)
Composition	-0.26^{***}	0.53^{***}
	(0.04)	(0.08)
Association	3.10^{***}	2.01^{***}
	(0.11)	(0.14)
Percent Explained	-9.15	20.86
Detailed Composition Components		
Family Structure	-0.06^{***}	-0.08^{***}
	(0.01)	(0.02)
Mother Age at 1st Birth	-0.12^{***}	-0.15^{***}
	(0.02)	(0.02)
Parental Education	0.00	0.13^{***}
	(0.02)	(0.03)
Parental Income	0.02	0.13^{***}
	(0.02)	(0.03)
Educational Attainment	0.06^{***}	0.22^{***}
	(0.02)	(0.03)
Work Experience	-0.16^{***}	0.28^{***}
	(0.02)	(0.05)

Table 1 continued

Detailed Association Component		
Family Structure	0.03	0.05
	(0.06)	(0.06)
Mother Age at 1st Birth	1.22*	1.10
	(0.50)	(0.66)
Parental Education	0.24	-0.16
	(0.12)	(0.14)
Parental Income	-0.48*	-0.06
	(0.22)	(0.24)
Educational Attainment	0.06	-0.05
	(0.09)	(0.11)
Work Experience	-0.03	-3.12**
	(0.69)	(0.96)
Constant	2.06*	4.25***
	(0.90)	(1.21)
N	9211	8963

Note: Statistical significance: * p < 0.05, ** p < 0.01, *** p < 0.001; Standard errors in parentheses.

Section V: GGP Sequences with State Alphabets Including Parental Home Leaving & Childless Cohabitation

Table 1: Oaxaca-Blinder Decompositions on Birth Cohort Differentials in Average Sequence Dissimilarity – Swedish GGP

	No Childless Cohabitation No Parental Home Leaving			Childless Cohabitation No Parental Home Leaving			Childless Cohabitation Parental Home Leaving		
	‘50s-‘60s	‘50s-‘70s	‘60s-‘70s	‘50s-‘60s	‘50s-‘70s	‘60s-‘70s	‘50s-‘60s	‘50s-‘70s	‘60s-‘70s
Difference	1.68***	5.20***	3.51***	0.86***	2.00***	1.13***	1.00***	2.12***	1.11***
	(0.26)	(0.29)	(0.29)	(0.17)	(0.19)	(0.20)	(0.11)	(0.15)	(0.15)
Composition	-0.00	0.69***	0.67***	0.07	0.47***	0.34***	0.14**	0.51***	0.32***
	(0.11)	(0.17)	(0.12)	(0.08)	(0.11)	(0.10)	(0.04)	(0.08)	(0.06)
Association	1.69***	4.51***	2.85***	0.80***	1.53***	0.79***	0.86***	1.61***	0.79***
	(0.25)	(0.31)	(0.29)	(0.16)	(0.18)	(0.19)	(0.11)	(0.15)	(0.14)
Percent Explained	0.00	13.26	19.08	8.13	23.50	30.08	14.00	24.05	28.82
Detailed Composition Component									
Family Structure	-0.13***	-0.06	-0.04	-0.05*	-0.03	-0.05+	-0.02	0.08*	0.03+
	(0.04)	(0.07)	(0.03)	(0.03)	(0.05)	(0.03)	(0.02)	(0.03)	(0.02)
Parental Education	0.17***	0.33***	0.17**	0.09**	0.08	0.04	0.05*	0.04	-0.01
	(0.04)	(0.10)	(0.06)	(0.03)	(0.08)	(0.04)	(0.02)	(0.05)	(0.02)
Educational Attainment	0.21***	0.48***	0.33***	0.12**	0.46***	0.30***	0.14***	0.37***	0.25***
	(0.06)	(0.09)	(0.07)	(0.04)	(0.07)	(0.06)	(0.03)	(0.05)	(0.04)
Gender	-0.25**	-0.06	0.20*	-0.08	-0.04	0.04	-0.03	0.01	0.05
	(0.08)	(0.10)	(0.10)	(0.06)	(0.06)	(0.06)	(0.03)	(0.04)	(0.04)

Table 1 continued

Detailed Association Component									
Family Structure	-1.77	-3.48*	-1.59*	1.19	0.36	-0.77	-0.62	-1.64*	-0.94*
	(1.56)	(1.52)	(0.79)	(1.26)	(1.11)	(0.56)	(0.74)	(0.72)	(0.38)
Parental Education	0.54	0.84	0.28	0.57	0.87*	0.25	0.56*	0.58*	0.02
	(0.56)	(0.56)	(0.47)	(0.35)	(0.38)	(0.31)	(0.25)	(0.26)	(0.26)
Educational Attainment	-0.28	0.41	0.63*	-0.12	0.10	0.26	-0.08	0.19	0.25+
	(0.25)	(0.33)	(0.30)	(0.17)	(0.21)	(0.20)	(0.10)	(0.15)	(0.14)
Gender	-0.09	-0.26	-0.18	-0.18	-0.14	0.03	-0.14	-0.40**	-0.27+
	(0.28)	(0.29)	(0.29)	(0.16)	(0.18)	(0.20)	(0.11)	(0.14)	(0.14)
Constant	3.29*	7.00***	3.71***	-0.67	0.35	1.02	1.14	2.87***	1.73***
	(1.66)	(1.57)	(0.95)	(1.32)	(1.15)	(0.73)	(0.81)	(0.80)	(0.49)
N	3146	2688	2828	3135	2666	2799	3141	2688	2815

Note: Statistical significance: * p < 0.05, ** p < 0.01, *** p < 0.001; Standard errors in parentheses.

Section VI: 5-Year Left & Right Truncated Sequences

Table 1: Oaxaca-Blinder Decompositions on Birth Cohort Differentials in Average Sequence Dissimilarity

	Men			Women		
	1952-1962	1952-1972	1962-1972	1952-1962	1952-1972	1962-1972
Difference	-0.62***	0.16	0.79***	-1.10***	-1.78***	-0.68***
	(0.12)	(0.13)	(0.12)	(0.15)	(0.13)	(0.12)
Composition	-0.12*	-0.01	0.08	-0.28***	-0.13	0.11*
	(0.05)	(0.08)	(0.05)	(0.08)	(0.09)	(0.05)
Association	-0.51***	0.17	0.70***	-0.82***	-1.65***	-0.79***
	(0.13)	(0.16)	(0.13)	(0.17)	(0.16)	(0.12)
Percent Explained	-9.68	1.53	5.12	10.64	12.30	11.95
Detailed Composition Component						
Family Structure	-0.05***	-0.08***	-0.03***	-0.12***	-0.15***	-0.04***
	(0.01)	(0.02)	(0.01)	(0.02)	(0.02)	(0.01)
Mother Age at 1st Birth	-0.05**	-0.08***	-0.01	-0.01	-0.08***	-0.01
	(0.02)	(0.02)	(0.01)	(0.02)	(0.02)	(0.01)
Parental Education	-0.06*	-0.03	-0.04	-0.04	0.02	-0.00
	(0.03)	(0.07)	(0.03)	(0.04)	(0.08)	(0.03)
Parental Income	-0.00	0.06	0.01	0.05	0.01	0.05*
	(0.03)	(0.05)	(0.02)	(0.04)	(0.07)	(0.02)
Educational Attainment	0.03	0.12***	0.08***	0.05*	0.13***	0.16***
	(0.02)	(0.03)	(0.02)	(0.02)	(0.04)	(0.03)
Work Experience	0.01	-0.00	0.08***	-0.21***	-0.06*	-0.04
	(0.02)	(0.01)	(0.02)	(0.06)	(0.02)	(0.02)
N	8676	9082	9802	8690	8697	9383

Note: Statistical significance: * $p < 0.05$, ** $p < 0.01$, *** $p < 0.001$; Standard errors in parentheses.

Section VII: S

Table 1: Summary Statistics of the Cohort-specific Populations and Analytical Samples used for Sequence Analysis and Oaxaca-Blinder Decompositions

	Population	Analytical Sample	
		Sequence Analysis	Decompositions
Distance			
1952		44.37	44.37
		(0.08)	(0.08)
1962		41.96	41.96
		(0.08)	(0.08)
1972		35.23	35.23
		(0.10)	(0.10)
Family Structure			
Single Parent			
1952	0.17	0.16	0.16
	(0.00)	(0.00)	(0.00)
1962	0.24	0.25	0.25
	(0.00)	(0.00)	(0.00)
1972	0.29	0.30	0.30
	(0.00)	(0.00)	(0.00)
Mother's Age at 1st Birth			
1952	24.55	24.55	24.55
	(0.02)	(0.05)	(0.05)
1962	23.49	23.47	23.47
	(0.01)	(0.05)	(0.05)
1972	23.41	23.38	23.38
	(0.01)	(0.04)	(0.04)
Parental Education			
(ref. Upper-Secondary)			
No Lower-Secondary			
1952	0.48	0.48	0.48
	(0.00)	(0.01)	(0.01)
1962	0.28	0.28	0.28
	(0.00)	(0.00)	(0.00)
1972	0.09	0.08	0.08
	(0.00)	(0.00)	(0.00)

Table 1, continued

Lower-Secondary			
1952	0.06	0.06	0.06
	(0.00)	(0.00)	(0.00)
1962	0.06	0.05	0.05
	(0.00)	(0.00)	(0.00)
1972	0.06	0.06	0.06
	(0.00)	(0.00)	(0.00)
Post-Secondary			
1952	0.13	0.12	0.12
	(0.00)	(0.00)	(0.00)
1962	0.23	0.22	0.22
	(0.00)	(0.00)	(0.00)
1972	0.35	0.34	0.34
	(0.00)	(0.00)	(0.00)
Parental Income			
(in 100,000 SEK)			
1952	3.13	3.11	3.11
	(0.01)	(0.02)	(0.02)
1962	4.02	4.01	4.01
	(0.01)	(0.02)	(0.02)
1972	4.62	4.64	4.64
	(0.01)	(0.02)	(0.02)
Educational Attainment			
(ref. Upper-Secondary)			
Lower-Secondary			
1952	0.23	0.24	0.24
	(0.00)	(0.00)	(0.00)
1962	0.15	0.15	0.15
	(0.00)	(0.00)	(0.00)
1972	0.08	0.08	0.08
	(0.00)	(0.00)	(0.00)

Table 1, continued

Secondary			
1952	0.29	0.27	0.27
	(0.00)	(0.00)	(0.00)
1962	0.30	0.29	0.29
	(0.00)	(0.00)	(0.00)
1972	0.43	0.43	0.43
	(0.00)	(0.00)	(0.00)
Work Experience			
1952	15.83	15.77	15.77
	(0.01)	(0.03)	(0.03)
1962	17.04	17.07	17.07
	(0.01)	(0.02)	(0.02)
1972	16.31	16.27	16.27
	(0.01)	(0.03)	(0.03)
Gender			
Women			
1952	0.50	0.50	0.50
	(0.00)	(0.01)	(0.01)
1962	0.49	0.50	0.50
	(0.00)	(0.01)	(0.01)
1972	0.48	0.48	0.48
	(0.00)	(0.01)	(0.01)
